# Targeting the activated microenvironment with endosialin (CD248)-directed CAR-T cells ablates perivascular cells to impair tumor growth and metastasis

**DOI:** 10.1136/jitc-2023-008608

**Published:** 2024-02-27

**Authors:** Sarah L Ash, Rebecca Orha, Holly Mole, Meg Dinesh-Kumar, Steven P Lee, Frances K Turrell, Clare M Isacke

**Affiliations:** 1 The Institute of Cancer Research, London, UK; 2 Department of Oncology, University of Lausanne, Lausanne, Switzerland; 3 University of Birmingham, Birmingham, UK; 4 Division of Cancer Sciences, Faculty of Biology, Medicine and Health, The University of Manchester, Manchester, UK

**Keywords:** Chimeric antigen receptor - CAR, Tumor microenvironment - TME, Breast Cancer, Lung Cancer

## Abstract

**Background:**

Targeting of solid cancers with chimeric antigen receptor (CAR)-T cells is limited by the lack of suitable tumor-specific antigens and the immunosuppressive, desmoplastic tumor microenvironment that impedes CAR-T cell infiltration, activity and persistence. We hypothesized that targeting the endosialin (CD248) receptor, strongly expressed by tumor-associated pericytes and perivascular cancer-associated fibroblasts, would circumvent these challenges and offer an exciting antigen for CAR-T cell therapy due to the close proximity of target cells to the tumor vasculature, the limited endosialin expression in normal tissues and the lack of phenotype observed in endosialin knockout mice.

**Methods:**

We generated endosialin-directed E3K CAR-T cells from three immunocompetent mouse strains, BALB/c, FVB/N and C57BL/6. E3K CAR-T cell composition (CD4^+^/CD8^+^ ratio), activity in vitro against endosialin^+^ and endosialin^–^ cells, and expansion and activity in vivo in syngeneic tumor models as well as in tumor-naive healthy and wounded mice and tumor-bearing endosialin knockout mice was assessed.

**Results:**

E3K CAR-T cells were active in vitro against both mouse and human endosialin^+^, but not endosialin^–^, cells. Adoptively transferred E3K CAR-T cells exhibited no activity in endosialin knockout mice, tumor-naive endosialin wildtype mice or in wound healing models, demonstrating an absence of off-target and on-target/off-tumor activity. By contrast, adoptive transfer of E3K CAR-T cells into BALB/c, FVB/N or C57BL/6 mice bearing syngeneic breast or lung cancer lines depleted target cells in the tumor stroma resulting in increased tumor necrosis, reduced tumor growth and a substantial impairment in metastatic outgrowth.

**Conclusions:**

Together these data highlight endosialin as a viable antigen for CAR-T cell therapy and that targeting stromal cells closely associated with the tumor vasculature avoids CAR-T cells having to navigate the harsh immunosuppressive tumor microenvironment. Further, the ability of E3K CAR-T cells to recognize and target both mouse and human endosialin^+^ cells makes a humanized and optimized E3K CAR a promising candidate for clinical development applicable to a broad range of solid tumor types.

**WHAT IS ALREADY KNOWN ON THIS TOPIC**

- The immunosuppressive tumor microenvironment and lack of suitable tumor-specific antigens has limited chimeric antigen receptor (CAR)-T cell efficacy against solid cancers, highlighting the need for the identification of new targets and approaches. Endosialin has limited expression in normal tissues but is strongly expressed by perivascular cells in solid tumor types, with a critical role in promoting tumor cell intravasation and metastatic dissemination.

**WHAT THIS STUDY ADDS**

- This study is the first to demonstrate the utility of targeting tumor-associated perivascular cells with CAR-T cells to limit tumor growth and metastasis, even in tumors with a cytotoxic T cell-excluded, immune cold phenotype. Further, as E3K CAR-T cells recognize mouse endosialin this allows for thorough assessment of CAR-T cell activity, tolerability and off-target and on-target/off-tumor effects in immunocompetent mice, in contrast to the majority of preclinical CAR-T cell studies performed in an immunodeficient setting.

**HOW THIS STUDY MIGHT AFFECT RESEARCH, PRACTICE OR POLICY**

- The therapeutic potential of endosialin-directed CAR-T cells is applicable to a wide range of solid tumor types and, due to the ability of the E3K CAR to recognize human as well as mouse endosialin, the opportunity for rapid clinical development. In addition, this study characterizes CAR-T cell products from different syngeneic mouse strains that will inform strain choices in future preclinical adoptive cell transfer studies.

## Introduction

Chimeric antigen receptor (CAR)-T cell therapy has revolutionized the treatment of hematological B cell malignancies, with six CAR-T cell therapies targeting either CD19 or B cell maturation antigen approved for clinical use to date. However, although recent positive outcomes in the GD2-CART01 neuroblastoma trial are encouraging,^1^ targeting non-hematological malignancies remains a challenge due, in large part, to the lack of tumor specific antigens and the desmoplastic and immunosuppressive tumor microenvironment (TME).^2^

CAR-T cell targeting of the microenvironment in solid tumors presents several advantages over direct tumor cell targeting.^3^ First, the genetic stability of stromal cells reduces the likelihood of antigen-loss mediated immune escape. Second, targeting the stromal cells that contribute to the physical and immunosuppressive barriers to cellular therapies minimizes the trajectory and energy expenditure of CAR-T cells required to reach their targets. Finally, many stromal components are common to a range of solid tumors, offering potential to benefit numerous cancer types. At present, the most documented stromal CAR-T cell target is fibroblast activation protein (FAP) upregulated on cancer-associated fibroblasts (CAFs). For example, in a highly desmoplastic preclinical pancreatic ductal adenocarcinoma (PDAC) model, CAR-T cell targeting of FAP^+^ CAFs removes the physical and immunosuppressive CAF barrier, and enhances tumor infiltration of other T cell subsets. This included a subsequent infusion of CAR-T cells targeting the PDAC tumor-associated antigen mesothelin, resulting in enhanced tumor control.^4^ Importantly a phase I trial of FAP^+^ mesotheliomas reported that FAP CAR-T cells administered intrapleurally were well tolerated.^5^

The transmembrane glycoprotein endosialin (CD248, TEM1) was originally identified as the most highly upregulated transcript in colorectal tumor vasculature compared with normal vasculature^6^ and subsequently demonstrated to be expressed exclusively by tumor-associated pericytes and perivascular CAFs in many solid tumor types with negligible or low level expression in healthy adult tissues.^7–11^ Functionally, endosialin binds to the vascular basement membrane protein multimerin-2 and contributes towards the maturation of the tumor-associated vasculature.^12^ Endosialin knockout mice display no overt phenotype, indicating that endosialin is not required for normal tissue homeostasis,^13^ but, when implanted with tumor cells, display an impairment in tumor progression and specifically in tumor cell dissemination.^14^ Therefore, we hypothesized that depleting endosialin^+^ cells would delay solid tumor progression by destabilizing the vasculature and hindering metastatic dissemination.

To target endosialin^+^ stromal cells, we developed a second generation CAR construct, E3K CAR. As immunological differences exist across immunocompetent mouse strains^15 16^ and strain-related differences in CAR-T cell tolerance have been reported^17 18^ we examined the phenotype and activity of E3K CAR-T cells derived from three independent strains, C57BL/6, BALB/c and FVB/N. We demonstrate depletion of endosialin^+^ stromal cells in subcutaneous and orthotopic tumors and delayed progression in syngeneic murine mammary carcinoma and lung cancer models, highlighting the potential of endosialin-targeting CAR-T cell therapy in multiple solid tumor types.

## Methods

### Cells and reagents

MCF7 (HTB-22), 10T1/2 (CCL-226), MRC-5 (CCL-171), 4T1 (CRL-2539, luciferase-tagged^19^ and LLC (CRL-1642) cells were from The American Type Culture Collection (ATCC). AT-3 and HRM1 cells^20^ were from Institute Pasteur de Lille and National Cancer Institute, respectively. Cells were maintained in Dulbecco's Modified Eagle Medium (DMEM), 10% FBS unless otherwise stated and routinely checked for *Mycoplasma* contamination (MycoAlert, Lonza), with identity of human cells confirmed by short tandem repeat (GenePrint 10 System, Promega). T cells were cultured in T cell media (TCM; Roswell Park Memorial Institute (RPMI), 10% FBS, 2 mM glutamine). Antibodies and dilutions are in online supplemental table 1.

10.1136/jitc-2023-008608.supp1 Supplementary data

### Endosialin monoclonal antibodies and E3K CAR-T cell generation

Monoclonal antibodies (mAbs) against endosialin were generated by immunizing rats with the 5 N-terminal domains of mouse endosialin fused to a human Fc domain. See online supplemental methods and online supplemental figure 2 for full details of the generation and characterization of rat anti-endosialin IgG_2A_-kappa mAbs 3K2L and 7A8F. Full details for the cloning strategy to generate the E3K CAR construct (based on mAb 3K2L) is provided in online supplemental methods and online supplemental table 2. The CAR construct was subcloned into the pMP71.tCD34.2A.CD19.IEVζ retroviral plasmid^21^ replacing the original scFv. The MP71 expression plasmid co-expresses a truncated human CD34 (hCD34) marker.^22^ Recombinant retrovirus generation and transduction of human peripheral blood mononuclear cells and mouse splenocytes was as previously described.^23^ Mouse splenocytes were isolated from 8-10 week-old female mice and T cells purified using a Ficoll Paque (GE HealthCare) density gradient.

#### In vivo studies

Female, 8–10 week-old mice were from Charles River. *Cd248^KO^* mice^24^ were backcrossed for >6 generations onto a BALB/c or C57BL/6 background. Health scores were assigned: 0, healthy, no signs of ill-health; −1, mild piloerection, behavior normal; −2, moderate piloerection and weight loss, behavior normal; −3, moderate piloerection, weight loss and other clinical signs (hunching, squinting, malaise). ARRIVE reporting guidelines^25^ were used with further details provided in online supplemental file 1. Mice were inoculated subcutaneously (flank) with 2.5×10^5^ 4T1 Luc or AT-3 cells in 50:50 phosphate-buffered saline (PBS): Matrigel, Corning 354230 or 5×10^5^ LLC cells in PBS; or orthotopically (fourth mammary fat pad) with 2×10^5^ HRM1 cells in PBS. For lymphodepletion assessment, tail vein blood was collected 18 hours after 4–5 Gy whole body X-irradiation. For adoptive cell transfer (ACT) experiments, tumor-bearing cohorts were assigned into tumor size-matched treatment groups. 4–5 Gy X-irradiation was performed, apart from NSG cohorts, 18 hours prior to tail vein injection of Mock or E3K CAR-T cells in Hanks' Balanced Salt Solution (HBSS). Where stated, DietGel and daily intraperitoneal injections (200 µL water) were administered for experiment duration. In ACT experiments using FVB/N mice, 1.33 mg/mL antibiotics (5:1 sulfamethoxazole: trimethoprim) was provided in the drinking water starting 2 days before X-irradiation until the end of the experiment. CAR-T cells were monitored by tail vein bleeds. At necropsy, blood was collected via cardiac puncture, heparinized and the serum separated by centrifugation.

#### Flow cytometry

Fibroblasts or tumor cells were detached (Cell Dissociation Buffer; Gibco 13151-014), incubated with CD16/32 Fc block (1:100, Invitrogen, 14-0161-85; 15 min), and stained in PBS+0.5% bovine serum albumin (BSA) at 4°C with rat anti-endosialin 3K2L antibody (30 min) and secondary antibody (20 min). Zombie Violet (1:1000, BioLegend, 423113) was used for live/dead discrimination. For analysis of circulating E3K CAR-T cells, 30 µL of heparinized tail vein blood was red blood cell-lysed (BD Pharm Lysis, BD Biosciences, 555899; 2× 4 min at 37°C). CAR-T cells were stained in 1% FBS in PBS (30 min at 4°C with antibodies against hCD34, mouse CD4 and mouse CD8, followed by 20 min incubation with secondary antibodies). 4',6-diamidino-2-phenylindole (DAPI) was used for live/dead discrimination. The number of CAR-T cells/mL blood was quantified using CountBright beads (C36950 or C36995, Invitrogen). Samples were run on the BD LSR II or FACSymphony A5 with compensation (UltraComp eBeads (eBioscience)), and data analysis on FlowJo V.10.

#### ELISA assays

ELISA assays were performed according to the manufacturer’s instructions (online supplemental table 3). For mouse interferon-γ (mIFN-γ) quantification in culture supernatant, target cells and T cells were cultured alone or at defined effector:target co-culture ratios in TCM for 24 hours. Samples not detected by the sensitivity of the assay were denoted as 0.

#### CAR-T cell cytotoxicity assay

Target cells were cultured alone or with T cells at defined effector:target co-culture ratios in TCM for 24–96 hours. T cells were removed with three PBS washes before target cell viability was assessed by CellTiter-Glo (Promega), with target cells alone defined as 100% viability.

#### Histology and immunofluorescence

3–4 µm sections were cut from formalin-fixed paraffin embedded tissues and H&E-stained. For immunofluorescence, sections were rehydrated and antigen-retrieved (DAKO antigen retrieval solution). Cells on coverslips were fixed (4% paraformaldehyde) and stained in PBS+1% BSA, 2% FBS at 4°C overnight (primary antibody) and 40 min at room temperature (secondary antibody), before incubation with Hoechst or DAPI. Slides were scanned using NanoZoomer Digital Pathology, Hamamatsu (low-power images). High-power images were taken with the Leica SP8 confocal microscope. Metastasis and necrosis were quantified manually, in a blinded fashion, from H&E-stained sections or by immunostaining for the tumor-cell marker Hmga2 for HRM1 metastases.^26^ Fiji software (ImageJ, 2.0.0-rc-54/1.51 hours) was used to determine the endosialin^+^ or endomucin^+^ area. Only viable tissue areas were quantified. Quantification of the percentage of endosialin^+^ blood vessels was performed on tumor sections costained for endomucin and endosialin, analyzing viable tissue only. The total number of vessels (endomucin^+^) and the number of endosialin^+^ vessels were quantified in eight ×20 randomly-chosen images.

### Immunohistochemistry of clinical samples

The tissue microarray of 245 invasive breast cancers (Breast Cancer Now Research Centre) and the immunohistochemical staining has been described previously.^27^ Staining with mAb B1/35^10^ and blinded scoring were performed.

#### Western blotting

Cells were lysed in radioimmunoprecipitation assay (RIPA) buffer and lysates sonicated for two cycles of 30 s. Blots were blocked with 5% milk before incubation with antibodies, and imaged and analyzed on a ChemiDoc System with Image Lab (Bio-Rad, V.6.1).

#### Statistics

Statistics were performed using GraphPad Prism V.8/9 software. Non-parametric tests were performed if analysis did not pass normality testing (Shapiro-Wilk). The following tests were used: two-tailed unpaired t*-*test or Mann-Whitney U test for comparisons between two groups; one-way analysis of variance (ANOVA) analysis with Tukey’s test for multiple comparisons or Kruskal-Wallis test with Dunn’s test for multiple comparisons when >2 groups were compared; two-way ANOVA followed by Sidak post hoc testing or three-way ANOVA followed by Tukey’s test for multiple comparisons if multiple groups with two or three variables were compared, respectively.

## Results

### Endosialin (*CD248*) expression is upregulated in the human and mouse tumor stroma

**Figure 1:**
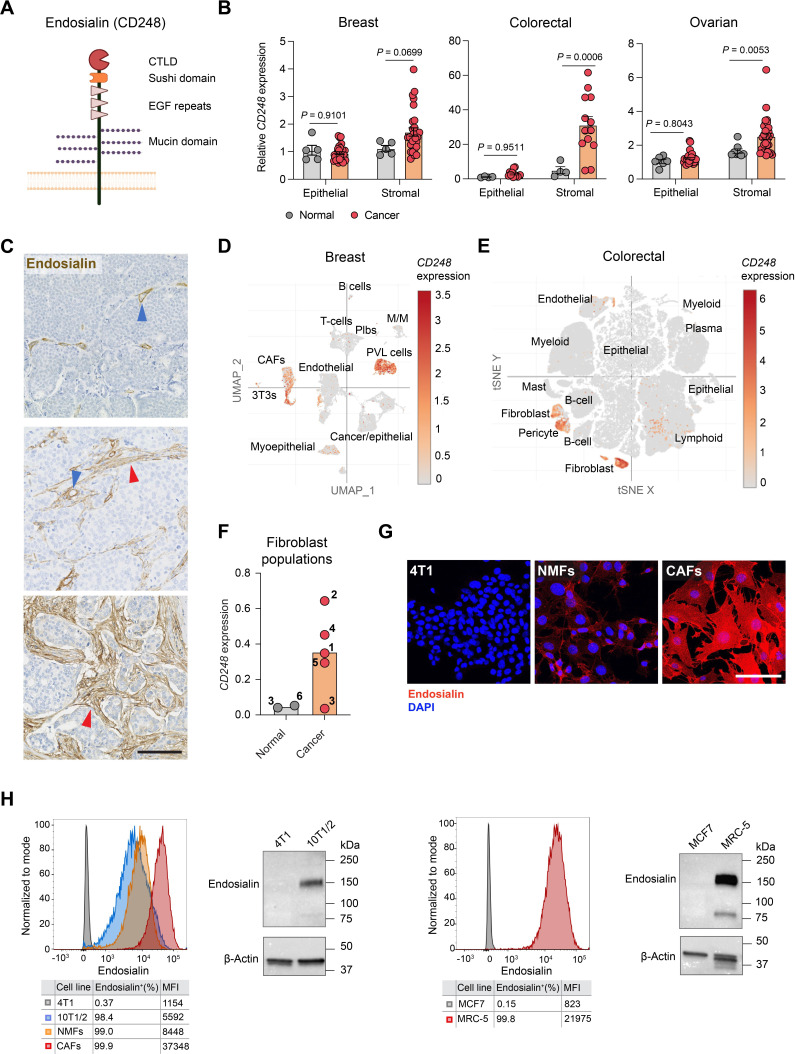
Upregulation of endosialin expression in the tumor stroma. (A) Structure of the endosialin (CD248) protein. (B) Endosialin (*CD248*) expression in stroma microdissected from tumors and normal tissues in breast (n=5 normal, n=28 cancer),^29^ colon (n=4 normal, n=13 cancer)^30^ or ovary (n=6 normal epithelial, n=8 normal stromal, n=32 cancer epithelial, n=31 cancer stromal).^31^ Data represent mean values±SEM, two-way ANOVA. (C) Representative images of invasive human breast cancers stained with anti-human endosialin mAb B1/35. Arrowheads indicate endosialin^+^ pericytes (blue) and CAFs (red). Scale bar, 125 µm. (D,E) Endosialin (*CD248*) expression in scRNA-seq data sets from one of three primary human breast cancers analyzed^32^ (panel D), and in a merged set of 62 colorectal cancers^33^ (panel E). (F) Cluster-specific endosialin (*CD248*) expression in fibroblast populations identified from scRNA-seq analysis of normal (adjacent non-malignant tissue) and cancer (tumor tissue) samples collected from patients with lung cancer.^36^ Each number represents an individual fibroblast cluster. (G) Mouse cells stained with anti-endosialin mAb 3K2L (red) and counterstained with DAPI (blue). Scale bar, 100 µm. Representative images are shown of two independent assays. (H) Flow cytometry and western blot analysis using mAb 3K2L of endosialin levels in mouse (left panel) and human (right panel) cells. Data is representative of two independent assays. ANOVA, analysis of variance; CAFs, cancer-associated fibroblasts; CTLD, C-type lectin-like domain; DAPI, 4′,6-diamidino-2-phenylindole; EGF, epidermal growth factor; mAb, monoclonal antibodies; MFI, median fluorescence intensity; NMF, normal mammary gland fibroblasts; PVL, perivascular-like; scRNA-seq, single-cell RNA sequencing; t-SNE, t-distributed stochastic neighbor embedding; UMPA, uniform manifold approximation and projection.

Endosialin is a ~175 kDa transmembrane receptor comprised of an N-terminal C-type lectin-like domain, a sushi domain, three epidermal growth factor repeats, a heavily O-glycosylated membrane proximal mucin domain, a single transmembrane domain and a short cytoplasmic tail (figure 1A). Our laboratory and others have previously reported that endosialin is expressed at low levels in normal adult tissues but highly expressed in the stroma of solid tumors.^7–11 28^ Consistent with these studies, in normal tissues of three different mouse strains endosialin is only detected on scattered fibroblasts with little or no expression on pericytes (online supplemental figure 1). Similarly, interrogation of gene expression profiling data of stroma microdissected from human malignant or normal breast,^29^ colon^30^ or ovary^31^ tissues demonstrated significantly upregulated *CD248* expression in the tumor stroma (figure 1B) while immunohistochemical analysis of a human invasive breast cancer tissue microarray revealed pronounced endosialin staining on pericytes in 184 out of 219 (84%) and on CAFs in 147 (67%) of the cases, (figure 1C). Single cell sequencing studies of breast^32^ and colon cancers,^33^ show endosialin (*CD248*) expression is largely restricted to perivascular-like cells and CAFs, with negligible expression on tumor cells or other stromal cell types (figure 1D,E). Moreover, single cell sequencing has revealed considerable heterogeneity in mesenchymal stromal cells identifying phenotypically and functionally distinct CAF/pericyte subpopulations.^34 35^ Comparing fibroblast populations in lung tumors and non-malignant lung tissue, Lambrechts and colleagues identified five CAF clusters with unique gene signatures and two normal fibroblast clusters. Expression of *CD248* is elevated in the CAF clusters, with the exception of CAF cluster 3 that overlaps with the normal fibroblast clusters (figure 1F). Notably, the CAF cluster with the highest *CD248* expression co-clusters with pericytes.^36^

Using the in-house generated mAb 3K2L (online supplemental methods and online supplemental figure 2), we confirmed that levels of endosialin protein are elevated in mouse CAFs compared with normal mouse mammary gland fibroblasts and are low or undetectable on mouse mammary tumor cell lines. In addition, flow cytometric analysis and western blotting demonstrated the ability of 3K2L to recognize both mouse and human endosialin (figure 1G,H), making it a promising candidate for development of CAR-T cells.

### Endosialin-directed E3K CAR-T cells exhibit specific activity in vitro

**Figure 2:**
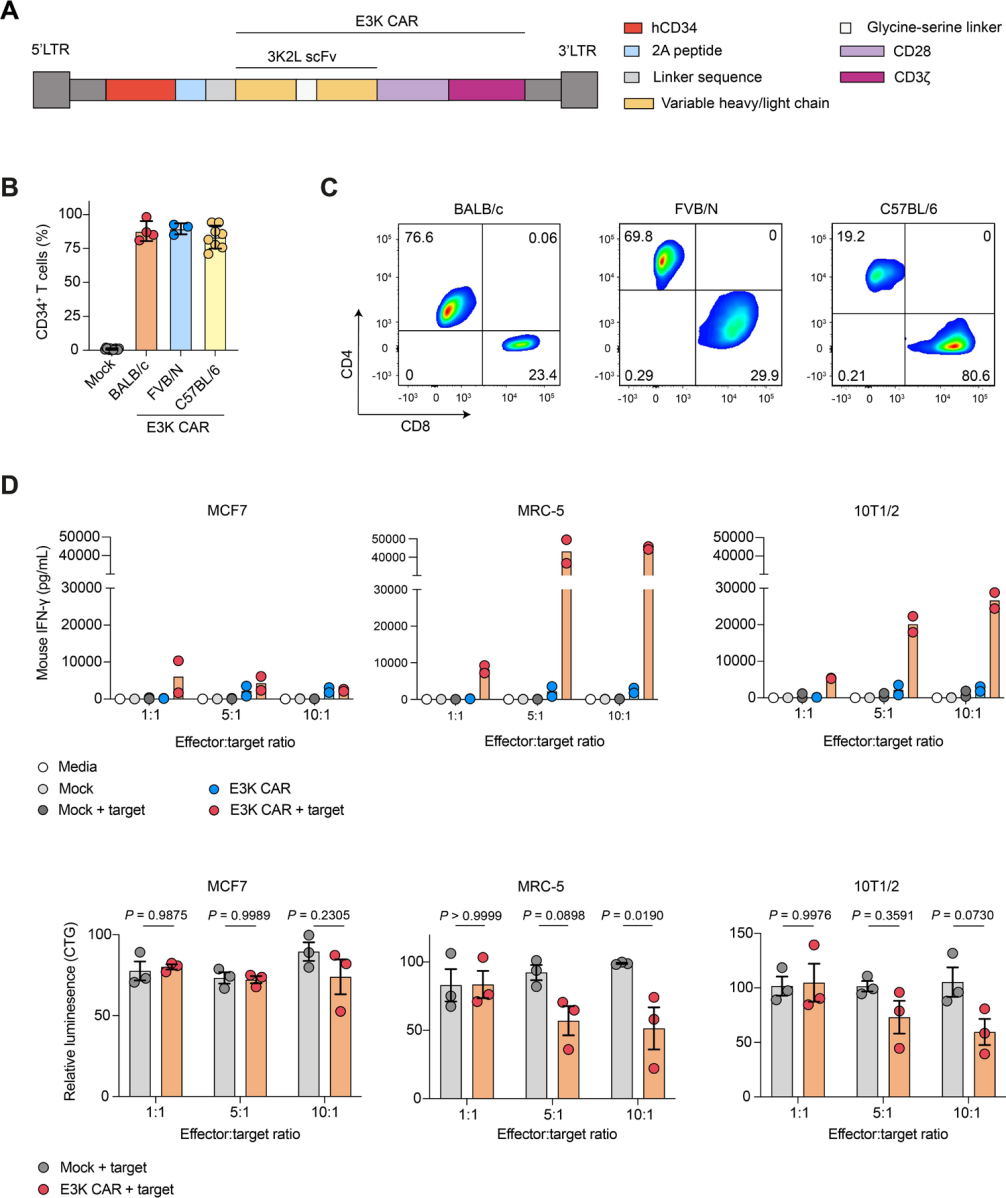
E3K CAR-T cells demonstrate specific activity against human and mouse endosialin^+^ fibroblasts. (A) Diagram of the E3K CAR construct. (B) Transduction efficiency of BALB/c (n=4), FVB/N (n=3) and C57BL/6 (n=8) E3K CAR-T cells determined by hCD34 expression in combined CD4^+^ and CD8^+^ T cell populations (mean values±SD). Data points represent independent transductions with strain-matched Mock-T cells used as hCD34^–^ controls. (C) Proportion of CD4^+^ and CD8^+^ E3K CAR-T cells in different mouse strains. Data is representative of all transductions shown in panel B. (D) E3K CAR-T cell activity in vitro. Mouse (2,000/well) or human (5,000/well) target cells were seeded in 96-well plates and the following day C57BL/6 Mock or E3K CAR-T cells were added at the indicated ratios. At 24 hours, mouse IFN-γ was detected in culture supernatants by ELISA (upper panels, n=2 independent assays). T cells were washed off and viability of target cells was measured by CellTiter-Glo with readouts from target cells cultured alone defined as 100% viability (lower panels; mean values±SD, two-way analysis of variance, n=3 independent assays). Data points represent independent assays with each assay performed with three technical replicates/condition. CAR, chimeric antigen receptor; hCD34, human CD34; IFN, interferon.

The scFv region of mAb 3K2L was cloned into a retroviral vector (pMP71) to encode a second-generation CAR construct, E3K CAR, in which the 3K2L scFv is linked to the human CD28 co-stimulatory domain and human CD3ζ cytoplasmic domain, together with a truncated hCD34 separated from the CAR by a cleavable foot-and-mouth disease virus 2A linker (figure 2A). Splenocytes from three different mouse strains (BALB/c, FVB/N and C57BL/6) were transduced with the E3K CAR construct, followed by lymphocyte isolation via density separation gradients. Flow cytometric analysis of the resulting cell population, in which CD8^+^ and CD4^+^ populations were gated together, revealed hCD34^+^ populations of >70% in all strains (figure 2B). Interestingly, C57BL/6 E3K CAR-T cells were majority CD8^+^, with an average CD4:CD8 ratio of 0.37 (range 0.24–0.56) whereas BALB/c and FVB/N E3K CAR-T cells were largely CD4^+^, with an average CD4:CD8 ratio of 2.1 (range 1.4–3.3) and 2.8 (range 1.9–4.3), respectively (figure 2C). E3K CAR-T cells retain the ability to bind endosialin (online supplemental figure 3A).

In in vitro assays both mouse (10T1/2) and human (MRC-5) endosialin^+^ stromal cell lines were used as target cells, alongside the human (MCF7) endosialin^–^ tumor cell line (figure 1H). Initial experiments were performed with C57BL/6 CAR-T cells due to their enrichment of CD8^+^ cytotoxic cells. Target cells were cultured alone or co-cultured with E3K CAR-T or mock transduced (Mock)-T cells at increasing effector:target ratios. Only E3K CAR-T cells co-cultured with endosialin^+^ stromal cells displayed elevated interferon (IFN)-γ release into the culture supernatants, with negligible levels of IFN-γ detectable when co-cultured with endosialin^-^ MCF7 cells (figure 2D upper panels). Similarly, in C57BL/6 mouse E3K CAR-T cell cytotoxicity assays, 10T1/2 and MRC-5 cell viability was significantly reduced at effector:target ratios of 5:1 or 10:1 compared with 10T1/2 and MRC-5 cells co-cultured with Mock-T cells or when E3K CAR-T cells were co-cultured with endosialin^–^ human MCF7 (figure 2D, lower panels) or mouse 4T1 (online supplemental figure 3B) tumor cells. These findings were not restricted to mouse CAR-T cells as human E3K CAR-T cells were specifically activated in response to wild-type mouse embryonic fibroblasts (MEFs) but not by endosialin^–^ CAL-51 tumor cells ectopically expressing human endosialin, nor by *Cd248*^KO^ MEFs or vector-alone transfected CAL-51 cells (online supplemental figure 4).

### E3K CAR-T cells limit metastasis of 4T1 tumors in immunocompromised mice

**Figure 3:**
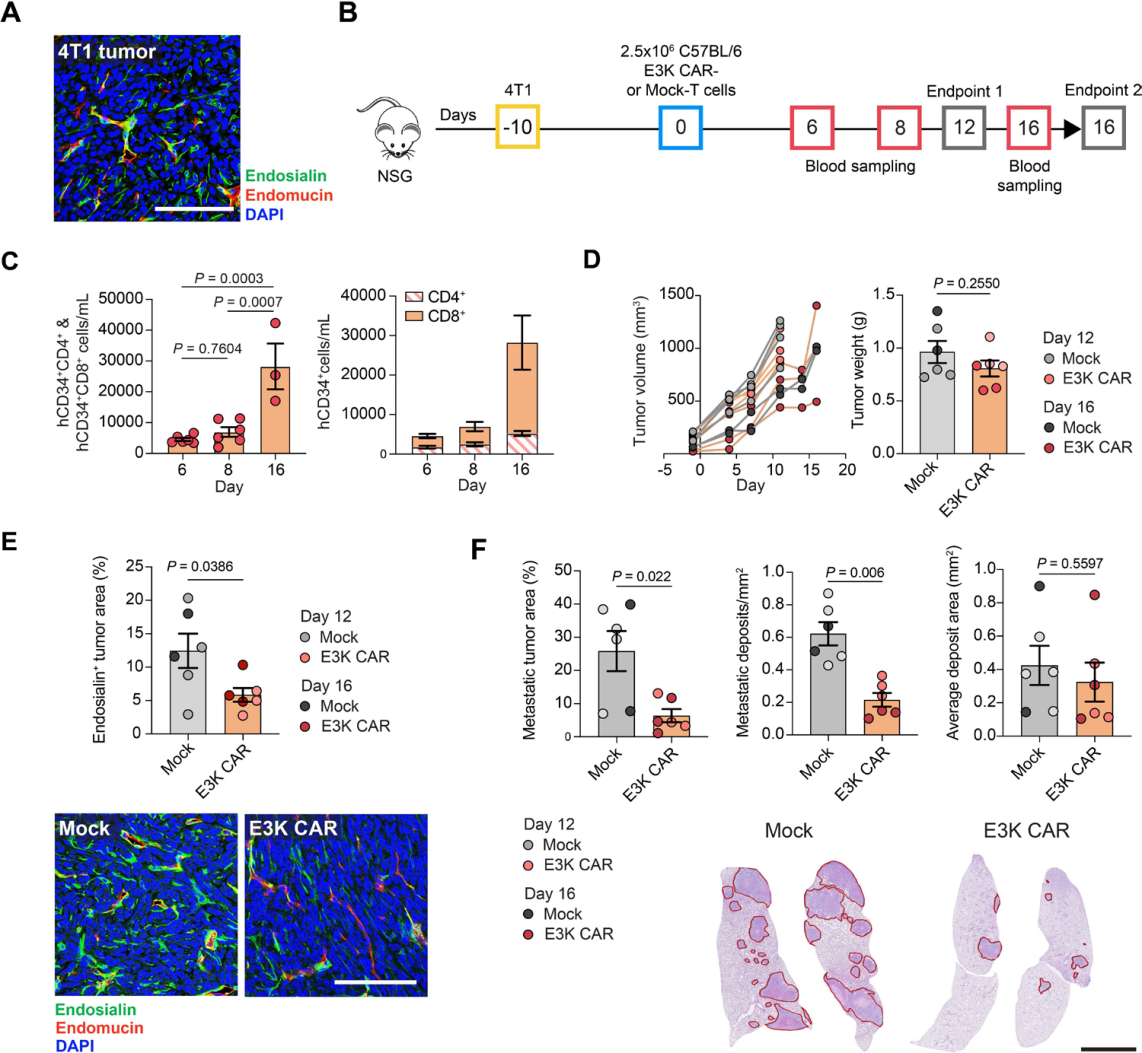
E3K CAR-T cells limit 4T1 tumor metastasis in NSG mice. (A) NSG mice were inoculated subcutaneously with 2.5×10^5^ 4T1 cells and sacrificed 13 days later. Shown is a representative image of a tumor section stained for endosialin (green), endomucin (red) and counterstained with DAPI (blue). Scale bar, 125 µm. (B) ACT experimental timeline. NSG mice were inoculated subcutaneously with 2.5×10^5^ 4T1 cells. Tumors were allowed to grow for 10 days to 30–200 mm^3^. Mice were inoculated intravenously with 2.5×10^6^ C57BL/6 Mock or E3K CAR-T cells (n*=*6 per group) and sacrificed on day 12 or day 16 post-ACT. (C) Number of circulating hCD34^+^CD4^+^ and hCD34^+^CD8^+^ E3K CAR-T cells monitored in venous blood (n*=*6, day 6 and 8; n*=*3, day 16). Left panel, hCD34^+^CD4^+^ and hCD34^+^CD8^+^ combined (mean values±SEM, one-way analysis of variance). Right panel, ratio of hCD34^+^CD4^+^ and hCD34^+^CD8^+^ cells (mean values±SEM). (D) Left panel, tumor growth curves for individual mice. Right panel, tumor weights at endpoint (mean values±SEM, unpaired t-test). (E) Tumor sections stained for endosialin (green), endomucin (red) and counterstained with DAPI (blue). Upper panel, percentage endosialin^+^ viable tumor area (mean values±SEM, unpaired t-test). Lower panel, representative tumor images from mice sacrificed on day 16. Scale bar, 125 µm. (F) Quantification of lung metastatic burden as per cent tumor area (left panel), number of metastases per mm^2^ (center panel) and average metastasis area (right panel) (mean values±SEM, unpaired t*-*test). Representative H&E-stained lung sections from mice sacrificed on day 16, red outlines indicate metastatic deposits. Scale bar, 2.5 mm. (D–F) Mice sacrificed on day 12 and day 16 are shown in light and dark circles, respectively. ACT, adoptive cell transfer; CAR, chimeric antigen receptor; DAPI, 4',6-diamidino-2-phenylindole; hCD34, human CD34.

The 4T1 murine mammary carcinoma cell line is a well characterized model known to spontaneously metastasize to the lungs and we previously reported a significantly reduced 4T1 metastasis in endosialin knockout (*Cd248*^KO^) mice.^14^ To investigate the ability of CD8^+^-heavy C57BL/6 E3K CAR-T cells to impair 4T1 metastasis, a pilot study was performed to determine the optimal timing of CAR-T cell treatment (online supplemental figure 5). NSG mice were inoculated subcutaneously with 4T1 cells and when tumors reached 200–300 mm^3^ in size (day 13), two animals were sacrificed with the remaining three mice sacrificed on day 20. Only two small metastatic lesions were detected in the two mice sacrificed on day 13 whereas the three mice sacrificed on day 20, when tumors were 800–1000 mm^3^, had a heavy metastatic burden throughout the lung tissue. Importantly, immunostaining of a day 13 primary tumor for endosialin and the endothelial marker endomucin revealed endosialin^+^ stromal cells throughout the tumor bed closely associated with the vasculature (figure 3A), indicating that the E3K CAR-T cell target is well expressed in developing tumors.

To assess the activity of E3K CAR-T cells in this model, 4T1-tumor bearing NSG mice were inoculated with C57BL/6 E3K CAR-T or Mock-T cells 10 days after tumor cell inoculation—a time chosen to ensure that ACT was given when the target was expressed but prior to metastatic outgrowth. Blood sampling was conducted 6, 8 and 16 days post-ACT and mice were sacrificed on day 12 or day 16 due to primary tumor size restrictions (figure 3B). hCD34^+^ E3K CAR-T cells were readily detectable in vivo, with an expansion observed by day 16 and the C57BL/6 CAR-T cell CD4:CD8 ratio maintained (figure 3C). There was no significant difference between the Mock and E3K CAR-T cell treated cohorts in terms of primary tumor growth or weight at necropsy (figure 3D), however, E3K CAR-T cell treated mice showed a significant ablation of endosialin^+^ cells in the primary tumor (figure 3E) and a significant reduction in overall metastatic burden and number of metastatic lesions (figure 3F). Interestingly there was no difference in the size of the metastatic lesions between the groups suggesting that, in this model, E3K CAR-T cells act primarily to reduce the dissemination of malignant cells from the primary tumor site.

### E3K CAR-T cells limit metastasis of 4T1 tumors in syngeneic BALB/c mice

**Figure 4:**
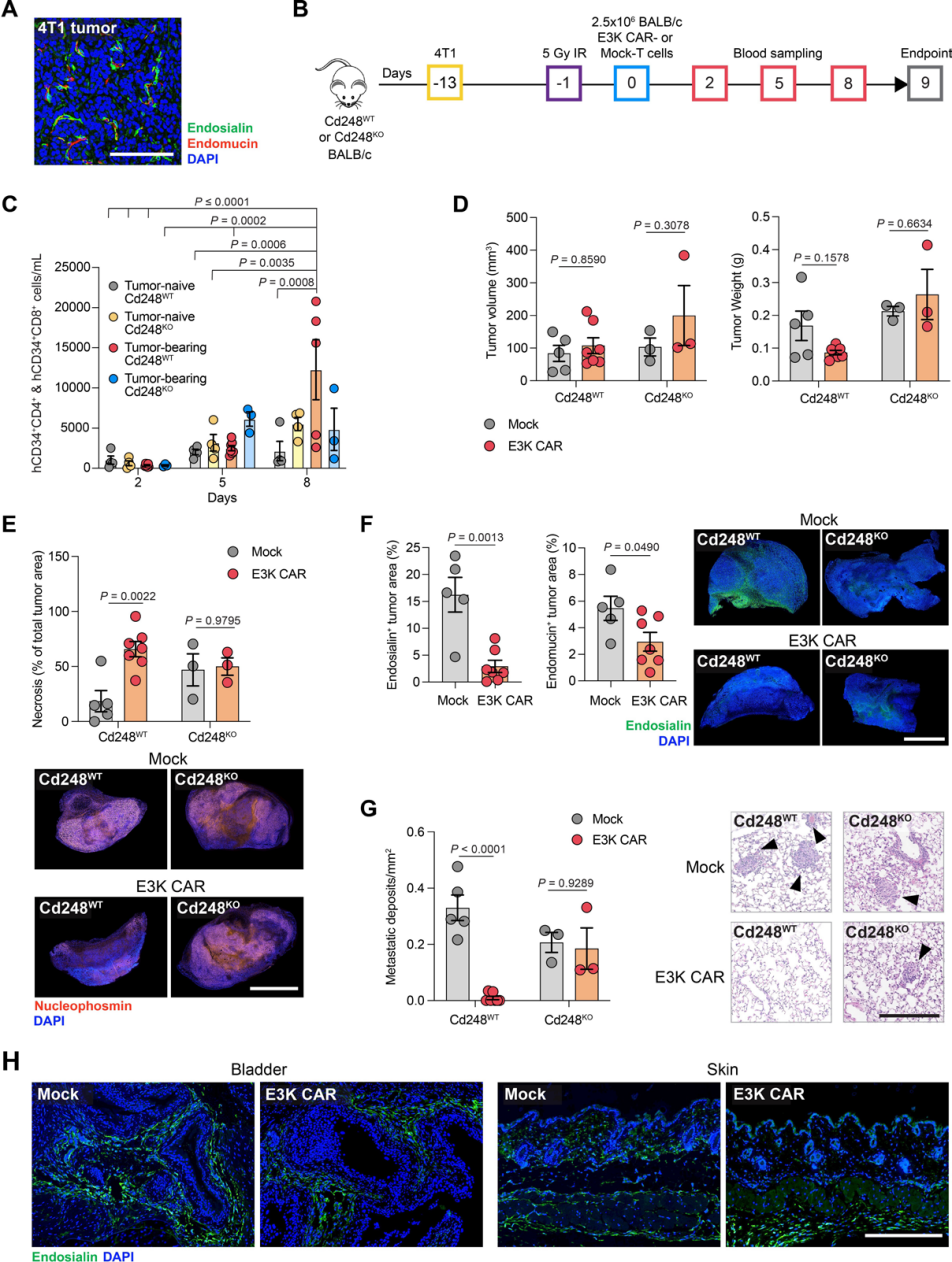
E3K CAR-T cells limit 4T1 tumor progression in BALB/c mice. (A) BALB/c mice were inoculated subcutaneously with 2.5×10^5^ 4T1 tumor cells and sacrificed 13 days later. Shown is a representative tumor section stained for endosialin (green), endomucin (red) and counterstained with DAPI (blue). Scale bar, 125 µm. (B) ACT experimental timeline. *Cd248*^WT^ and *Cd248*^KO^ mice were inoculated subcutaneously with 2.5×10^5^ 4T1 cells or left tumor-naive. After 12 days, when the tumors were 20–200 mm^3^ in volume, mice were subjected to 5 Gy whole body X-irradiation 18 hours prior to intravenous injection with 2.5×10^6^ BALB/c Mock or E3K CAR-T cells. Mice were sacrificed 9 days post-ACT. *Cd248*^WT^ tumor-bearing (Mock n*=*5, E3K CAR n*=*7), *Cd248*^WT^ naive (n*=*4); *Cd248*^KO^ tumor-bearing (n*=*3), *Cd248*^KO^ naive (n*=*4). (C) Circulating combined hCD34^+^CD4^+^ and hCD34^+^CD8^+^ CAR-T cells monitored in venous blood. Samples from Mock-T cell treated mice were used as negative controls (mean values±SEM, three-way ANOVA). (D) Tumor volume (left panel) and tumor weight (right panel) at endpoint (mean values±SEM, two-way ANOVA). (E) Upper panels, tumor necrosis quantified from H&E-stained section (mean values±SEM, two-way ANOVA). Lower panels, representative images of tumor sections stained for the cell viability marker nucleophosmin illustrating loss of tumor cell viability in E3K CAR-T cell treated *Cd248*^WT^ mice. Scale bar, 2.5 mm. (F) Quantification endosialin^+^ (left panel) and endomucin^+^ (center panel) areas in viable tumor tissue (mean values±SEM, unpaired t-test). Right panel, representative images of tumor sections showing absence of endosialin staining in *Cd248*^KO^ mice and loss of endosialin staining in E3K CAR-T cell treated *Cd248*^WT^ mice. Scale bar, 2.5 mm. (G) Quantification of lung metastatic burden from H&E-stained sections (mean values±SEM, two-way ANOVA). Right panel, representative images, arrowheads indicate metastatic lesions. Scale bar, 250 µm. (H) Representative images of bladder and skin sections from *Cd248*^WT^ tumor-bearing mice treated with Mock or E3K CAR-T cells and stained for endosialin (green) and counterstained with DAPI (blue). Scale bar, 250 µm. ACT, adoptive cell transfer; ANOVA, analysis of variance; CAR, chimeric antigen receptor; DAPI, 4',6-diamidino-2-phenylindole; hCD34, human CD34.

Given the strong anti-metastatic phenotype observed against 4T1 tumors in NSG mice treated with E3K CAR-T cells, we next addressed whether this efficacy could be translated into the syngeneic BALB/c setting. BALB/c E3K CAR-T cells were characterized in vitro, demonstrating endosialin-specific CAR-T cell activation and cytotoxicity against both human and mouse target cell viability (online supplemental figure 6). It has been reported that efficient lymphodepletion prior to ACT in syngeneic mouse models greatly enhances the engraftment of adoptively transferred cells.^37^ To this end, a small cohort of BALB/c mice were subject to 5 Gy whole body X-irradiation or left unirradiated. After 18 hours, venous blood was collected for flow cytometry analysis, confirming substantial lymphodepletion in irradiated mice (online supplemental figure 7A). A preliminary study was then performed in 4T1 tumor-bearing or tumor-naive BALB/c mice, using the experimental timeline outlined in online supplemental figure 7B, with BALB/c E3K CAR or Mock-T cells injected 13 days after 4T1 cell inoculation when the tumors were small but had strong endosialin expression in the tumor stroma (figure 4A). Flow cytometry analysis of blood samples collected 5 and 8 days post-ACT revealed a greater expansion of E3K CAR-T cells in tumor-bearing mice compared with tumor-naive mice (online supplemental figure 7C). Further, a trend towards reduced tumor growth (online supplemental figure 7D), a reduction in endosialin^+^ cells within the tumors (online supplemental figure 7E) and, a striking reduction in the number of metastatic lung deposits was observed in the E3K CAR-T cell treated cohort, with 70% of E3K CAR-T cell treated mice having no detectable lung disease (online supplemental figure 7F). However, though promising, this experiment was terminated 9 days post-ACT due to weight loss (online supplemental figure 7G) and declining health status in the E3K CAR-T cell treated cohort.

To further characterize the antitumor effects, as well as any associated toxicity, of E3K CAR-T cells in the BALB/c model, a second study was performed using tumor-bearing and tumor-naive mice and with *Cd248*^KO^ mice as additional controls (figure 4B). In this subsequent study, food accessibility and hydration were enhanced in an attempt to alleviate toxicity symptoms however, as before, the mice were sacrificed early (day 9 post-ACT) due to declining health status in the *Cd248*^WT^, tumor-bearing, E3K CAR-T cell treated cohort, with no adverse effects observed in the other cohorts (online supplemental figure 8A). E3K CAR-T cells expanded in *Cd248*^WT^ tumor-bearing mice and persisted at constant levels in all other groups (figure 4C). As in the preliminary experiment, at this early endpoint there were no significant differences in primary tumor volume or weight at necropsy between the groups (figure 4D). By contrast, when compared with the Mock-T cell-treated *Cd248*^WT^ mice, tumors in E3K CAR-T-treated *Cd248*^WT^ mice displayed a significant increase in tumor necrosis (figure 4E), likely due to depletion of endosialin^+^ cells and loss of endomucin^+^ endothelial cells in the tumor stroma (figure 4F), and significantly reduced metastatic burden (figure 4G). In the *Cd248*^KO^ mice treatment with E3K CAR-T cells had no impact on tumor necrosis or metastatic burden, with immunostaining confirming the lack of endosialin expression (figure 4E–G). In post-mortem analysis the notable difference in the tumor-bearing *Cd248*^WT^ E3K CAR-T cell treated BALB/c mice compared with all other groups was presence of pale livers with darkened gallbladders, with livers showing abnormal histology in H&E staining (online supplemental figure 8B,C). In the clinic, the most common adverse event associated with CAR-T cell therapy is cytokine release syndrome (CRS), characterized by elevated serum interleukin (IL)-6 levels. When compared with Mock-T cell treated controls, circulating IL-6 was significantly elevated in tumor-bearing *Cd248*^WT^, E3K CAR-T cell treated mice (online supplemental figure 8D). In a separate cohort of *Cd248*^WT^ 4T1 tumor-bearing mice, E3K CAR-T cell treated mice were paired with Mock-T cell treated controls and pairs were sacrificed when the E3K CAR-T cell treated mouse displayed signs of toxicity. Again, serum IL-6 was significantly elevated in E3K CAR-T cell treated mice, although with lower concentrations than observed in the previous experiment. Measurement of additional serum inflammatory markers associated with CRS revealed a significant elevation in serum ferritin, but not tumor necrosis factor-α (TNF-α) (online supplemental figure 8E). It has previously been reported that patients with macrophage activation syndrome (MAS)-like manifestations following CAR-T cell therapy have elevated serum ferritin, but decreased serum C reactive protein (CRP).^38^ Here, we observed decreased serum CRP in E3K CAR-T cell treated mice, suggesting that E3K CAR-T cell associated toxicity is linked to macrophage activation. Further, MAS has been reported to affect liver function and histology.^39 40^

The lack of CAR-T cell activity in the tumor-bearing *Cd248*^KO^ mice and against endosialin^−^ cells indicated that toxicity did not result from off-target cross-reactivity. Although endosialin expression is strongly upregulated in the tumor stroma, scattered endosialin^+^ fibroblasts are detected in normal tissues (online supplemental figure 1), however, no visible differences in endosialin-staining were observed in bladder and skin tissues between E3K CAR and Mock-T cell treated tumor-bearing *Cd248*^WT^ mice (figure 4H). Similarly, in a BALB/c mouse wound healing assay, which results in the accumulation of endosialin^+^ cells during wound closure, treatment with E3K CAR-T cells had no impact on the rate of wound closure, did not result in any notable depletion of endosialin^+^ cells at the wound site or in normal skin on the opposite flank and did not result in any toxicities (online supplemental figure 9A–D). These findings indicate that E3K CAR-T cells have little or no on-target, off-tumor CAR-T cell activity and that the observed toxicities were related to on-target, on-tumor activity.

### E3K CAR-T cells exhibit antitumor efficacy and are well-tolerated in syngeneic FVB/N and C57BL/6 mammary tumor models

**Figure 5:**
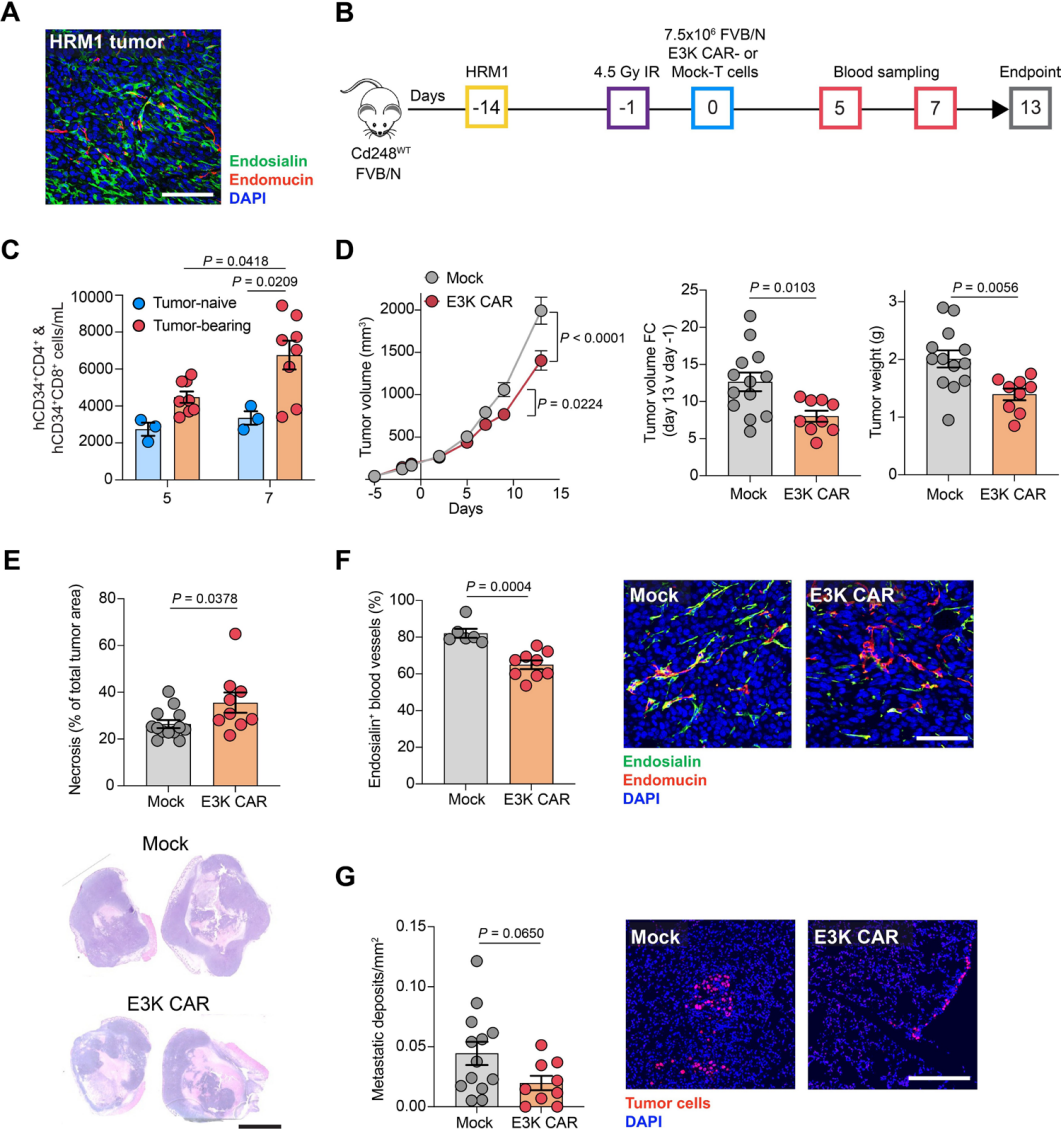
E3K CAR-T cells limit HRM1 tumor growth and metastasis in FVB/N mice. (A) FVB/N mice were inoculated orthotopically with 2×10^5^ HRM1 tumor cells and sacrificed 14 days later. Shown is a representative tumor section stained for endosialin (green), endomucin (red) and counterstained with DAPI (blue). Scale bar, 100 µm. (B) ACT experimental timeline. *Cd248*^WT^ FVB/N mice were inoculated orthotopically with 2×10^5^ HRM1 cells or left tumor-naive. After 13 days when the tumors were 80–280 mm^3^ in volume, mice were subjected to 5 Gy whole body X-irradiation 18 hours prior to intravenous injection with 7.5×10^6^ Mock or E3K FVB/N CAR-T cells. Mice were sacrificed 13 days post-ACT. Tumor-bearing (Mock n=13, E3K CAR n=9), tumor-naive (Mock n=3, E3K CAR n=3). (C) Circulating combined hCD34^+^CD4^+^ and hCD34^+^CD8^+^ CAR-T cells monitored in venous blood from a subset of mice (mean values±SEM, two-way ANOVA). (D) Left panel, tumor growth curves (mean values±SEM, two-way ANOVA); center panel, fold change in tumor volume from day −1 to endpoint (mean values±SEM, unpaired t*-*test); right panel, tumor weight at endpoint (mean values±SEM, unpaired t*-*test). (E) Quantification of tumor necrosis from H&E-stained sections (mean values±SEM, unpaired t-test). Lower panel, representative H&E-stained sections. Scale bar, 5 mm. (F) Percent endosialin^+^ blood vessels quantified in a subset of tumors stained for endosialin (green), endomucin (red) and counterstained with DAPI (blue) (mean values±SEM, Mann-Whitney U test). Right panel, representative images. Scale bar, 100 µm. (G) Quantification of lung metastatic burden in sections stained for the tumor cell marker Hmga2 (red) and counterstained with DAPI (blue) (mean values±SEM, unpaired t-test). Right panel, representative images. Scale bar, 250 µm. ACT, adoptive cell transfer; ANOVA, analysis of variance; CAR, chimeric antigen receptor; DAPI, 4',6-diamidino-2-phenylindole; hCD34, human CD34.

To determine whether the toxicities observed in the 4T1-tumor bearing mice is specific to the BALB/c background and to test the E3K CAR-T cells in independent models we first assessed CAR-T cell activity in FVB/N mice inoculated with the ER^+^ HRM1 mouse mammary carcinoma cells.^26^ BALB/c and FVB/N mice have a similar CD4-heavy CD4:CD8 T cell ratio (figure 2C) and FVB/N and BALB/c E3K CAR-T cells have comparable activity against endosialin^+^ cells in vitro (online supplemental figures 6 and 10). Like 4T1 tumors,^41^ HRM1 tumors have abundant endosialin^+^ pericytes and CAFs (figure 5A). We confirmed that whole-body X-irradiation in FVB/N mice resulted in lymphodepletion (online supplemental figure 11A) and performed a pilot experiment (online supplemental figure 11B) using 2.5–10×10^6^ FVB/N Mock-T and E3K CAR-T cells to identify a safe and efficacious E3K CAR-T cell dose. E3K CAR-T cells persisted with evidence of expansion 7–13 days post-ACT in tumor-bearing mice and decreased tumor growth following E3K CAR-T cell treatment was observed (online supplemental figure 11C,D). None of the mice exhibited signs of E3K CAR-T cell toxicity (online supplemental figure 11E). Given these results, we proceeded to treat cohorts of HRM1 tumor-bearing mice or non-tumor-bearing controls with 7.5×10^6^ FVB/N E3K CAR-T or Mock-T cells (figure 5B). Again, in tumor-bearing mice, E3K CAR-T cells persisted and expanded in vivo 5–7 days post-ACT (figure 5C), accompanied by a significant decrease in primary tumor growth and weight at endpoint (figure 5D), a significant increase in tumor necrosis (figure 5E), depletion of endosialin^+^ pericytes within the tumor stroma (figure 5F) and a trend for decreased metastases in E3K CAR-T cell treated mice (figure 5G). As with the pilot experiment, there was no evidence of E3K CAR-T cell-mediated toxicity with the mouse weights for all groups remaining stable and no detectable abnormalities in the organs at necropsy (online supplemental figure 11F,G).

**Figure 6:**
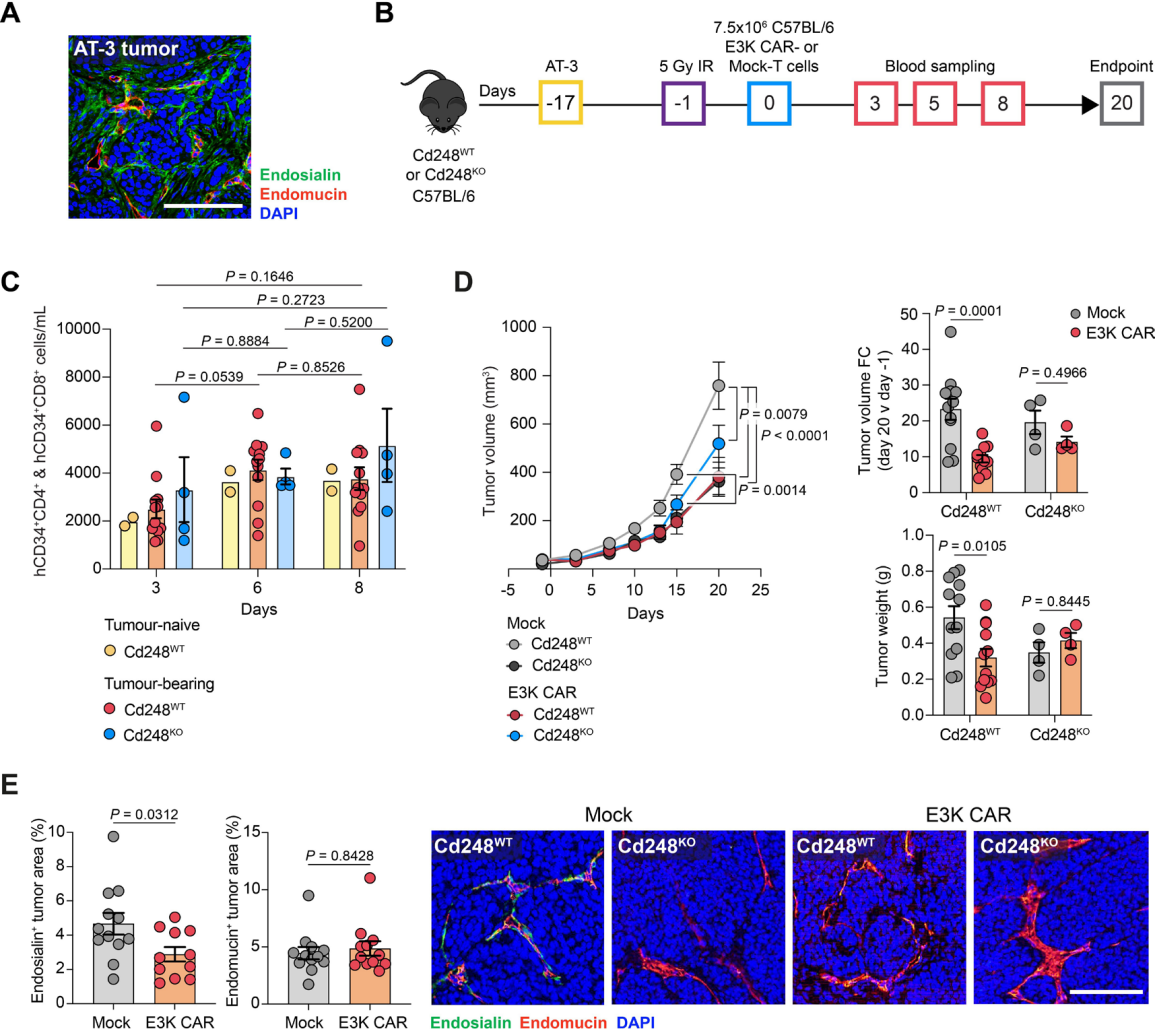
E3K CAR-T cells limit AT-3 tumor growth in C57BL/6 mice. (A) C57BL/6 mice were inoculated subcutaneously with 2.5×10^5^ AT-3 tumor cells and sacrificed 17 days later. Shown is a representative tumor section stained for endosialin (green), endomucin (red) and counterstained with DAPI (blue). Scale bar, 125 µm. (B) ACT experimental timeline. *Cd248*^WT^ and *Cd248*^KO^ mice were inoculated subcutaneously with 2.5×10^5^ AT-3 cells or left tumor-naive. After 16 days, when the tumors were 20–100 mm^3^, mice were subjected to 5 Gy whole body X-irradiation 18 hours prior to injection with 7.5×10^6^ Mock or C57BL/6 E3K CAR-T cells. Mice were sacrificed on day 20 post-ACT. *Cd248*^WT^ tumor-bearing (Mock n*=*12, E3K CAR n*=*12), *Cd248*^WT^ naive (Mock n=2, E3K CAR n=2); *Cd248*^KO^ tumor-bearing (Mock n*=*4, E3K CAR n*=*4). (C) Circulating combined hCD34^+^CD4^+^ and hCD34^+^CD8^+^ CAR-T cells monitored in venous blood (mean values±SEM, two-way ANOVA). (D) Left panel, tumor growth curves (mean values±SEM, two-way ANOVA); upper right panel, fold change in tumor volume from day −1 to endpoint; lower right panel, tumor weight at endpoint. For right panels, data shows mean values±SEM, two-way ANOVA. (E) Tumor sections were stained for endosialin (green), endomucin (red) and counterstained with DAPI (blue). Quantification of endosialin^+^ (left panel) and endomucin^+^ (center panel) staining in viable tumor tissue (mean values±SEM). Left panel, unpaired t-test; an outlier (with a value of 15.01%) was identified via outlier analysis in GraphPad Prism (ROUT method, *q*=1%) and excluded from the analysis. Center panel, Mann-Whitney U test. Right panel, representative images of tumor sections showing lack of endosialin staining in *Cd248*^KO^ mice and loss of endosialin staining in E3K CAR-T cell treated *Cd248*^WT^ mice. Scale bar, 125 µm. ACT, adoptive cell transfer; ANOVA, analysis of variance; CAR, chimeric antigen receptor; DAPI, 4',6-diamidino-2-phenylindole; hCD34, human CD34.

Next, we examined a C57BL/6 model inoculating mice with the poorly metastatic MMTV-PyMT tumor-derived AT-3 murine mammary carcinoma line. As in the other models, early stage (day 17) AT-3 tumors display strong endosialin staining on pericytes and closely associated CAFs, however, in contrast to other models which display a chaotic vasculature with immature vessels, the vascular architecture in AT-3 tumors is dominated by large well-structured vessels (figure 6A). Having confirmed that 5 Gy X-irradiation leads to effective lymphodepletion in C57BL/6 mice (online supplemental figure 12A), a pilot study was conducted (online supplemental figure 12B) in which AT-3 tumor-bearing C57BL/6 mice were treated with Mock-T cells (1×10^7^, n=2) or one mouse each received 2.5, 5 or 7.5×10^6^ E3K CAR-T cells. All doses were well tolerated, with body weights remaining stable throughout the study and no signs of toxicity (online supplemental figure 12C). There was an indication of reduced tumor growth with doses of 5×10^6^ or greater E3K CAR-T cells (online supplemental figure 12D). Following these promising results, we conducted a full study to assess E3K CAR-T cell efficacy against AT-3 tumor progression in C57BL/6 *Cd248*^WT^ and *Cd248*^KO^ mice (figure 6B). Interestingly, in all mice E3K CAR-T cells persisted but did not expand (figure 6C). *Cd248*^WT^ mice treated with E3K CAR-T cells showed a significant decrease in primary tumor growth and tumor weight at necropsy in (figure 6D) and a significant depletion of endosialin^+^ cells, but not endomucin^+^ endothelial cells, within the tumor stroma (figure 6E) indicating that in the AT-3 tumors where the vessels are larger and more mature compared with the 4T1 tumors, depletion of endosialin^+^ pericytes is insufficient to disrupt the vascular architecture. No evidence of CAR-T cell toxicity was observed in any cohort and, as seen in the HRM1-FVB/N model, examination of the tissues at necropsy revealed healthy livers in all groups (online supplemental figure 12E,F) with no evidence of CAR-T cell-mediated depletion of fibroblasts in healthy tissue (online supplemental figure 12G). E3K CAR-T cells had no impact on primary tumor growth or tumor weight in *Cd248*^KO^ mice, supporting the on-target, on-tumor specificity of E3K CAR-T cells.

### E3K CAR-T cells limit lung carcinoma metastatic progression in C57BL/6 mice

**Figure 7:**
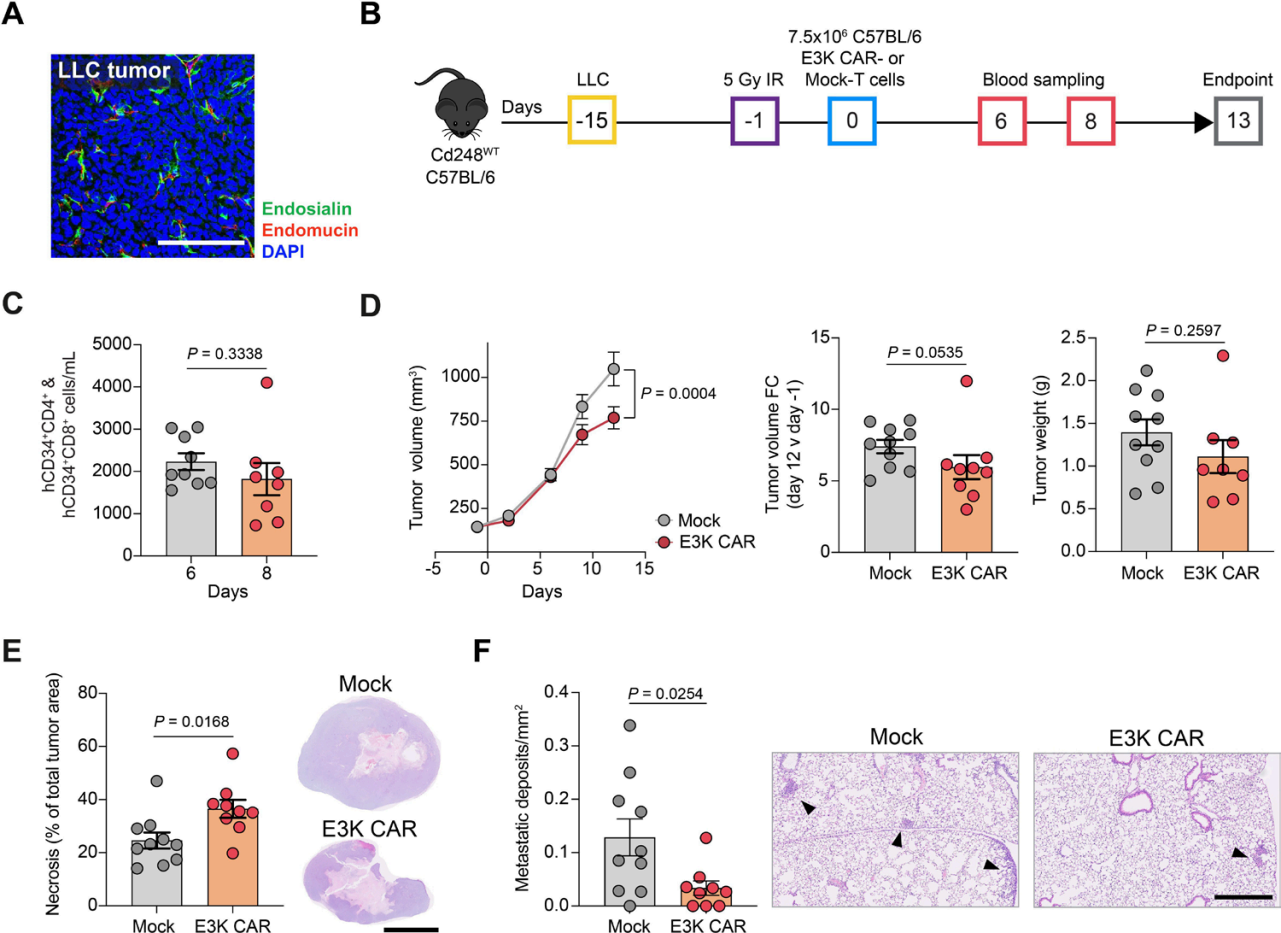
E3K CAR-T cells limit Lewis lung carcinoma tumor growth and metastasis in C57BL/6 mice. (A) C57BL/6 mice were inoculated subcutaneously with 5×10^5^ LLC tumor cells and sacrificed 15 days later. Shown is a representative tumor section stained for endosialin (green), endomucin (red) and counterstained with DAPI (blue). Scale bar, 125 µm. (B) ACT experimental timeline. *Cd248*^WT^ C57BL/6 mice were inoculated subcutaneously with 5×10^5^ LLC cells. After 14 days, when the tumors were 100–200 mm^3^, mice were subjected to 5 Gy whole body X-irradiation 18 hours prior to injection with 7.5×10^6^ C57BL/6 Mock (n=10) or E3K CAR (n=9) T cells. Mice were sacrificed on day 13 post ACT. (C) Circulating combined hCD34^+^CD4^+^ and hCD34^+^CD8^+^ CAR-T cells monitored in venous blood from a subset of mice (mean values±SEM, unpaired t-test). (D) Left panel, tumor growth curves (mean values±SEM, two-way analysis of variance; center, fold change in tumor volume from day −1 to endpoint; right panel, tumor weight at endpoint. For center and right panels, data shows mean values±SEM, Mann-Whitney U test (center), unpaired t-test (right). (E) Quantification of tumor necrosis from H&E-stained sections (mean values±SEM, unpaired t*-*test) and representative tumor sections. Scale bar 5 mm. (F) Quantification of lung metastatic lesions in H&E-stained sections (mean values±SEM, unpaired t-test) and representative images. Arrowheads indicate metastatic deposits. Scale bar, 500 µm. ACT, adoptive cell transfer; CAR, chimeric antigen receptor; DAPI, 4',6-diamidino-2-phenylindole; hCD34, human CD34; LLC, Lewis lung carcinoma.

Finally, to confirm that E3K CAR-T cell activity is not restricted to breast cancer models, we examined a second C57BL/6 tumor model, the Lewis lung carcinoma (LLC) model, reported by us and others^14 42^ to spontaneously metastasize to the lungs. Immunostaining of LLC tumors collected at 14 days post tumor cell implantation demonstrated a chaotic vasculature consisting mainly of immature vessels, with abundant endosialin^+^ pericytes (figure 7A). Following the schedule outlined in figure 7B, C57BL/6 *Cd248*^WT^ mice bearing LLC tumors were subject to 5 Gy whole body X-irradiation followed 18 hours later by ACT of E3K CAR-T or Mock-T cells. Mice were sacrificed 13 days post-ACT due to tumor size restrictions. None of the mice exhibited signs of toxicity (online supplemental figure 13). As with the AT-3 model, E3K CAR-T cells persisted but did not expand in vivo (figure 7C) driving a significant decrease in primary tumor growth, a trend towards decreased tumor weight at necropsy (figure 7D) associated with a significant increase in primary tumor necrosis (figure 7E) and a significant decrease in the lung metastatic burden (figure 7F).

Together, mutiple breast and lung cancer models demonstrate that E3K CAR-T cells are active in impairing tumor progression in multiple mouse strains and, importantly, that toxicities are restricted to the 4T1 tumor-bearing BALB/c mice with no toxicities observed in the FVB/N and C57BL/6 models despite mice being infused with 3× greater number of CAR-T cells.

## Discussion

The challenges of targeting solid tumors with CAR-T cells are well documented and have resulted in alternative CAR-T cell strategies being explored.^2 3^ The approach taken here is to target cells in the tumor microenvironment, with perivascular cells being of particular interest due to their vascular localization such that extravasating CAR-T cells will immediately access their target and not have to navigate the dense immunosuppressive stroma. Endosialin presents an exciting target due to its strong upregulation on pericytes and CAFs in the tumor stroma, its lack of expression on pericytes associated with normal tissue vasculature and the absence of phenotype associated with the *Cd248*^KO^ mouse. In this study, CAR-T cells based on two rat anti-endosialin mAbs 3K2L and 7A8F were generated (online supplemental methods, online supplemental figure 2). In preliminary experiments mAb 7A8F based CAR-T cells were inferior to the E3K CAR-T cells and not pursued further. Encouragingly, transfusion of E3K CAR-T cells in healthy, tumor-naive animals was well-tolerated, with no indication of normal tissue targeting or targeting of cells within wound healing sites, suggesting that the density of off-tumor endosialin or its distance from the vasculature is insufficient for CAR-T cell recruitment and activation.

Using multiple preclinical models we demonstrate that E3K CAR-T cells are active against endosialin-expressing cells in vitro and within the tumor stroma. This is in contrast to a previous study where the activity of CAR constructs containing different anti-endosialin scFvs was described as modest and was not pursued in animal models,^43^ making our study the first to demonstrate activity of endosialin directed CAR-T cells in vivo. Conflicting reports have been published concerning the effects of pericyte or CAF ablation on tumor progression. In some cases, depletion of these cells has resulted in accelerated disease progression,^44–46^ however we did not observe this phenomenon. On the contrary, we observed significant delays in tumor progression in four syngeneic models. Of these, the 4T1, HRM1 and LLC models are immunologically “cold” characterized by few infiltrating cytotoxic T cells.^47–49^ Despite this, E3K CAR-T cells were effective against these tumors, supporting the notion that targeting pericytes circumvents the physical and immunosuppressive barriers to CAR-T cell activity. A limitation of the present study was the inability to track E3K CAR-T cells in the tumor environment due to the lack of antibodies against hCD34 that work in formalin-fixed paraffin-embedded (FFPE) material. Investigating the spatial distribution of E3K CAR-T cells would provide information as to whether the observed effects are solely due to targeting of perivascular cells or whether the CAR-T cells also target CAFs positioned more distally from vascular structures. Further, characterization of the E3K CAR-T cells that have homed to the tumor will be important to determining whether they would benefit from next generation engineering,^50^ such as ablating *TGFBR2* to prevent T cell exhaustion in the transforming growth factor-β (TGF-β)-rich TME^51^ or co-expressing IL-7 and CCL19 to enhance infiltration and survival.^52^

In this study, E3K CAR-T cell target specific toxicity was observed in 4T1 tumor-bearing BALB/c mice whereas no toxicity was observed in tumor-bearing C57BL/6 or FVB/N mice treated with equivalent or greater doses of CAR-T cells. BALB/c strain specific CAR-T cell toxicity has been reported by others^18^ and has been suggested to result from the higher proportion of CD4^+^ CAR-T cells in this strain.^17^ In the present study, BALB/c derived E3K CAR-T cells were indeed found to be majority CD4^+^, however this was also true of FVB/N E3K CAR-T cell products. In both BALB/c and FVB/N models, E3K CAR-T cells were observed to expand in the circulation but with greater expansion in the BALB/c model. In the clinic, onset of CAR-T cell-related toxicities often correlates with peak expansion in the circulation and higher expansion levels are associated with more severe toxicity.^53 54^ Therefore, it is possible that greater E3K CAR-T cell expansion is, at least in part, responsible for the toxicity observed in the BALB/c model. To date, no correlation between the number of CD4^+^ CAR-T cells and toxicity severity has been observed in the clinic. On the contrary, a correlation between the number of CD8^+^ CAR-T cells and CRS severity has been observed in patients.^55^ This suggests that the toxicity observed in BALB/c mice may be specific to this murine strain and, although further investigation is required, that the liver phenotype and serum profiling in the 4T1-BALB/c model indicate an MAS-like event. An increasing number of CAR-T cell therapies are now translated into the clinic armed with a suicide switch allowing depletion of CAR-T cells should toxicity arise. For example, in the recent GD2-CART01 trial, the CAR-T cells express an inducible caspase 9 suicide gene which was activated in one patient with successful CAR-T cell depletion.^1^ Alternatively, integration of an ON or OFF switch CAR design has been demonstrated in preclinical models to allow titratable remote control of CAR-T cell function, alleviating toxicities associated with excessive cytokine secretion or on-target off-tumor activity.^56 57^

To investigate activity in the tumor stroma, we focused primarily on models of breast cancer. Primary breast cancers are usually surgically resected however the first line therapy may be neoadjuvant chemotherapy to downsize the tumor prior to surgery. E3K CAR-T cell therapy may offer an alternative or complement to neoadjuvant therapy to limit disease progression in these patients. However, our data would suggest that endosialin targeting will also be effective in other tumor types that require more invasive surgery, such as lung cancers, and against established metastatic disease that has recruited a functional vasculature. In addition to targeting the tumor stroma, endosialin expression is upregulated in tumor cells of mesenchymal origin such as soft tissue sarcomas and osteosarcomas.^58–60^ Further studies will be required to establish if the optimal value of E3K CAR-T cells, whether for targeting the tumor stroma or endosialin^+^ tumors, will be in combination with other therapies.

In summary, E3K CAR-T cells represent a promising approach to targeting the tumor stroma and limiting metastatic spread with negligible effects on normal tissue. In addition, the ability of the E3K CAR to recognize both mouse and human endosialin makes it an ideal construct for humanization and optimization, and further therapeutic development. Finally, due to the wide range of solid tumor types that display upregulated endosialin expression in the tumor stroma, E3K CAR-T cells have the potential to benefit a wide range of patients with cancer.

10.1136/jitc-2023-008608.supp2 Supplementary data

## Data Availability

Data are available upon reasonable request.
