# Supplementary material for: Targeting the activated microenvironment with endosialin (CD248)-directed CAR-T cells ablates perivascular cells to impair tumor growth and metastasis
Source: J Immunother Cancer. 2024 Feb 27;12(2):e008608. doi: 10.1136/jitc-2023-008608 (PMC10900351; doi:10.1136/jitc-2023-008608)
Supplement: Supplementary data [file jitc-2023-008608supp001.pdf]

Targeting the activated microenvironment with endosialin (CD248) directed CAR-T cells ablates perivascular cells to impair tumor growth and metastasis

Sarah L. Ash, Rebecca Orha, Holly Mole, Meg Dinesh-Kumar, Steven P. Lee, Frances K. Turrell and Clare M. Isacke

SUPPLEMENTARY MATERIAL

Supplementary Methods

mAb generation and characterization
CAR construct generation and transduction of T cells
Human IFN-γ ELISA
In vivo studies

Supplementary Tables

Supplementary Table 1 | Antibodies
Supplementary Table 2 | RT-PCR primers for CAR construct generation
Supplementary Table 3 | ELISA kits

Supplementary Figures 1 - 9

Supplementary Figure 1 | Endosialin expression in healthy adult mouse tissues.
Supplementary Figure 2 | Characterization of the rat anti-endosialin 3K2L and 7A8F antibodies.
Supplementary Figure 3 | E3K CAR-T cells bind endosialin and exhibit no activity against endosialin$^-$ 4T1 cells
Supplementary Figure 4 | Human E3K CAR-T cells recognize and are activated by mouse and human endosialin$^+$, but not endosialin$^-$, cells.
Supplementary Figure 5 | 4T1 tumor growth in NSG mice.
Supplementary Figure 6 | *In vitro* characterization of BALB/c E3K CAR-T cells.
Supplementary Figure 7 | Characterization of E3K CAR T-cell activity in a syngeneic BALB/c model.
Supplementary Figure 8 | E3K CAR-T cell associated toxicity in BALB/c mice is limited to *Cd248*^{WT}, 4T1 tumor-bearing mice.
Supplementary Figure 9 | E3K CAR-T cells exhibit no activity and are not associated with toxicity in wound healing assays.
Supplementary Figure 10 | *In vitro* characterization of FVB/N E3K CAR-T cells.
Supplementary Figure 11 | Characterization of E3K CAR-T cell activity against HRM1 tumors in FVB/N mice.
Supplementary Figure 12 | Characterization of E3K CAR-T cell activity against AT-3 tumors in C57BL/6 mice.
Supplementary Figure 13 | E3K CAR-T cells show no toxicity in LLC3 tumor-bearing mice.

References

Ash SL, *et al. J Immunother Cancer* 2024; 12:e008608. doi: 10.1136/jitc-2023-008608

Supplemental Methods

mAb generation and characterization

Male PVG rates were immunized with the mouse endosialin-human Fc fusion protein (**Supplementary Figure 2A,B**) using the following schedule: Day 1 and Day 28 (immunization of the Peyer's patches (0.1 ml @ 2 mg/ml); Day 56 (Intraperitoneal immunization (0.1 ml @ 2 mg/ml); Day 84 (immunization of the Peyer's patches (0.1 ml @ 2 mg/ml); Day 87 (harvest spleen). Splenocytes were fused Y3Ag1.2.3 rat myeloma cells and supernatants from the resulting hybridomas screened by ELISA against the immunizing mouse endosialin-Fc fusion protein. Positive hybridomas were cross-screened against an irrelevant human Fc fusion protein to identify and discard hybridomas producing antibody against the human Fc domain. Remaining positive hybridomas were single cell cloned and screened for their ability to recognized full-length mouse endosialin ectopically expressed in endosialin-negative human MCF-7 cells, resulting in the identification of two positive clones 3K2L and 7A8F (**Supplementary Figure 2C**). 3K2L and 7A8F were both determined to be IgG_{2A}-kappa antibodies using the Raybio Rapid Rat Ig Isotyping Array (AAR-ISO-G1). Antibodies were purified from hybridoma supernatants on HiTrap Protein G columns (Sigma, GE17-0404-01).

The specificity of mAbs 3K2L and 7A8F and their ability to recognize endogenous mouse endosialin was confirmed by immunoblotting against 10T1/2 mouse pericyte-like cells transduced with either non-targeting control shRNAs or an shRNA against mouse endosialin (**Supplementary Figure 2D**). The ability of mAb 3K2L to recognize both mouse and human endosialin was assessed in the current study (**Figure 1H**).

CAR construct generation

RNA was extracted from the 3K2L and 7A8F hybridomas and reverse transcribed, a poly-C tail added to the 3' ends and cDNA amplified using a poly-G forward primer (primer pGI-TdT) and reverse primers specific for either the rat IgG_{2A} heavy chain constant region (primer rtIGHG1/2A) or the rat kappa light chain constant region (primer rtIGKC). A second nested PCR was then performed using a pGI-TdT anchor (primer pGI-TdT anchor) and nested primers for heavy (primer rtIGHG1/2A nest) and light (primer rtIGKC nest) chains. All custom oligonucleotides were obtained from Sigma (**Supplementary Table 2**). Amplified sequences were used to construct an scFv (comprising the variable heaving and variable light regions joined by a flexible Gly-Ser linker), from which gBlocks were synthesized (Integrated DNA Technologies, Leuven). An existing MP71 CAR-expression plasmid[1] was cleaved using Not1/Cla1 to remove the existing scFv and replaced with the 7A8F/3K2L-derived scFv

gBlocks. In preliminary experiments, the 7A8F-based CAR T cells were inferior to the 3K2L-based E3K CAR T cells and not pursued (data not shown).

Human IFN-γ ELISA

Maxisorp plates (Thermo Fisher; 443404) were coated with 50 µL/well anti-human IFN-γ antibody (Thermo Fisher; M700A) in 0.1M Na_2HPO_4 pH 9 at 0.75 µg/mL and left for 12 hours at 4°C. All subsequent incubations were performed at room temperature. Plates were incubated for 2 hours with blocking buffer (1% BSA/PBS, 0.05% Tween) and then washed four times with PBS/0.05% Tween. Test supernatants and IFN-γ standards (40000 pg/mL to 31.25 pg/mL) were added at 50 µL/well for 3 hours, before plates were washed 4 times with PBS/0.05% Tween and 50 µL/well biotinylated anti-IFN-γ antibody (Thermo Fisher; M701B, 0.375 µg/mL in blocking buffer) was added for 1 hour. Plates were then washed another 4 times and incubated for 30 minutes with 50 µL/well extrAvidin peroxidase (1/1000 with blocking buffer; Sigma). After a further 8 wash steps, 50 µL/well TMB substrate (Life Technologies, Frederick USA) was added for 20 minutes before the reaction was stopped with 1 M phosphoric acid (50 µL/well). Absorbance was measured at 450 nm on a plate reader, subtracting background absorbance values read at 655nm.

In vivo studies

All animal work was carried out at the Institute of Cancer Research. Animal holding rooms were maintained within the parameters recommended in the Home Office Code of Practice with temperatures being 21°C ± 2 °C, humidity 55% ± 10% and a light cycle of 12 hours dark/light. For all studies, mice were acclimatized for a minimum of 5 days, protocols were not registered before the start of the experiment and the experimental unit was the individual animal, each independently allocated to a treatment group. Mice were not randomized but assigned treatment groups based on tumor-size to ensure that before ACT, groups were matched for average tumor size. Investigators were aware of group allocation during the allocation and conduct of the experiment, and some outcome assessment (tumor size, mouse health) but other outcome assessment (metastasis and target expression) and data analysis was performed blinded. The outcome measures assessed were CAR-T cell expansion, primary tumor size, necrosis, target expression, metastasis and mouse health.

The number of mice/group is stated in the corresponding figure legend. The sample size for adoptive cell transfer (ACT) experiments was n=5-13 for tumor-bearing mice and was calculated based on how variable tumor growth was for the particular model and previous experience with spontaneous metastasis assays performed in-house, with the exception of

some *Cd248*^{KO} groups where 3-4 mice/group were used due to mouse availability. For tumor-naive (ACT, lymphodepletion, wound-healing, tumor growth, endosialin expression) cohorts the sample size was smaller (n=2-4) due to the absence of variability in tumor-growth. A total of 213 mice were used in this study. For studies to determine CAR-T cell dose, there was n=1-2 mice/group before the full ACT experiment was performed once the optimal dose had been established.

All controls were age-matched and, for ACT experiments, matched for tumor-size. For lymphodepletion studies, non-irradiated, tumor-naive mice were used as controls. For ACT studies, Mock-T cell-treated tumor-bearing mice were used as controls (unless otherwise stated in pilot or wound-healing studies when control mice received HBSS only), with tumor-naive *Cd248*^{WT} and tumor-bearing *Cd248*^{KO} mice used as additional control cohorts where stated. For studies to assess tumor growth and endosialin-positivity there was no control cohort as the experiment was performed to determine the timing of ACT for future experiments or the level of endosialin in the stroma, respectively, and so no control group was needed.

With the exceptions listed below, no mice were excluded in the analysis. Mice were sacrificed before treatment allocation if their tumors grew intraperitoneally. An outlier was identified in figure 6E (see figure legend for statistical test) and excluded from the analysis.

ACT experiments (dose-determining studies included) were repeated with equivalent results as follows: 4T1 model (5 independent experiments- 4 in BALB/c mice, 1 in NSG mice); HRM1 model in FVB/N mice (3 independent experiments); AT-3 in C57BL/6 mice (2 independent experiments). ACT in the LLC model was only performed once.

Licence endpoints were reached if a mouse showed continued signs of ill-health, exhibited weight loss of 20% over 72 hours or if the primary tumor (unilateral) reached a mean diameter of >17 mm. The maximum tumor size permitted by the Licence (mean diameter 18 mm) was not breached.

Ash SL, *et al. J Immunother Cancer* 2024; 12:e008608. doi: 10.1136/jitc-2023-008608

**Supplementary Table 1 |** Antibodies					
**Antibody Target**	**Source**	**ID**	**Species**	**Application**	**Dilution**
Endosialin (P13)	Isacke lab	MacFadyen et al., 2007	Rabbit	IHC	1/500
Endosialin (3K2L; 1 mg/mL stock)	Isacke lab	MacFadyen et al., 2007	Rat	WB<br>Flow	1/500<br>1/100
Endosialin (B1/35)	Isacke lab	MacFadyen et al., 2005	Mouse	IHC<br>WB	0.2 µg/mL<br>0.2 µg/mL
Endomucin (V7C7)	Santa Cruz	sc65495	Rat	IHC	1/1000
Nucleophosmin	Invitrogen	32-5300	mouse	IHC	1/2000
HMG2A	ThermoFisher	PA521320	Rabbit	IHC	1/300
CD4-PE	Biolegend	100408	Rat	Flow	1/100
CD8-FITC	Biolegend	10076	Rat	Flow	1/100
CD34-APC	Biolegend	343608	Mouse	Flow	1/20
β-actin	Sigma	A5441	Mouse	WB	1/25000
Mouse IgG-488	Invitrogen	A11001	Goat	Flow	1/1000
Mouse IgG-555	Invitrogen	A21127	Goat	Flow	1/1000
Human IgG-488	Invitrogen	A10631	Mouse	Flow	1/100
Rat IgG-555	Invitrogen	A21434	Goat	Flow	1/1000
Rabbit IgG-488	Invitrogen	A11034	Goat	IHC	1/1000
Rat IgG-HRP	Abcam	ab205720	Goat	WB	1/5000
Mouse IgG-HRP	Abcam	ab205719	Goat	WB	1/2000
APC, allophycocyanin; Flow, Flow cytometry; HRP, horseradish peroxidase; IgG, immunoglobulin G; PE, phycoerythrin, WB, western blot					

**Supplementary Table 2** | RT-PCR primer for CAR construct generation (obtained from Sigma)		
Primer Name	Target	**Sequence**
pGI-TdT	Poly C sequence.	ACG GTG CAA ACC TTC CTC CAA ATC GGG IIG GGI IGG GII
rtIGHG1/2A	Outer primer for rat IgG_{2A} heavy chain constant region.	TCC CAG GGT CAC CAT GGA GTT AC
rtIGKC	Outer primer for rat kappa light chain constant region.	GAT ACA CGA CTG WGG CAC CTC CAG T
pGI-TdT anchor	Anchor for pGI TdT primer	ACG GTG CAA ACC TTC CTC CAA ATC GGG
rtIGHG1/2A nest	Inner primer for rat IgG_{2A} heavy chain	GTC ACC ATG GAG TTA CTT TTG AGA GCA GT
rtIGKC nest	Inner primer for rat kappa light chain constant region.	GGA AGA TRG ATA CAG TTG GTG CAG CAT C
Degenerate bases: I = deoxyinosine; W = A or T; R = A or G		

Supplementary Table 3 | ELISA kits

**Target analyte**	**Source**	**ID**
mIFN-γ	Abcam	ab100689
mIL-6	Abcam	ab100712
mFerritin	Abcam	ab157713
mCRP	BioTechne	MCRP00
mTNFα	BioTechne	MTA00B

mIFN-γ, mouse interferon-γ; mIL-6, mouse interleukin-6; mCRP, mouse C-reactive protein; mTNFα, mouse tumor necrosis factor α

Ash SL, *et al. J Immunother Cancer* 2024; 12:e008608. doi: 10.1136/jitc-2023-008608

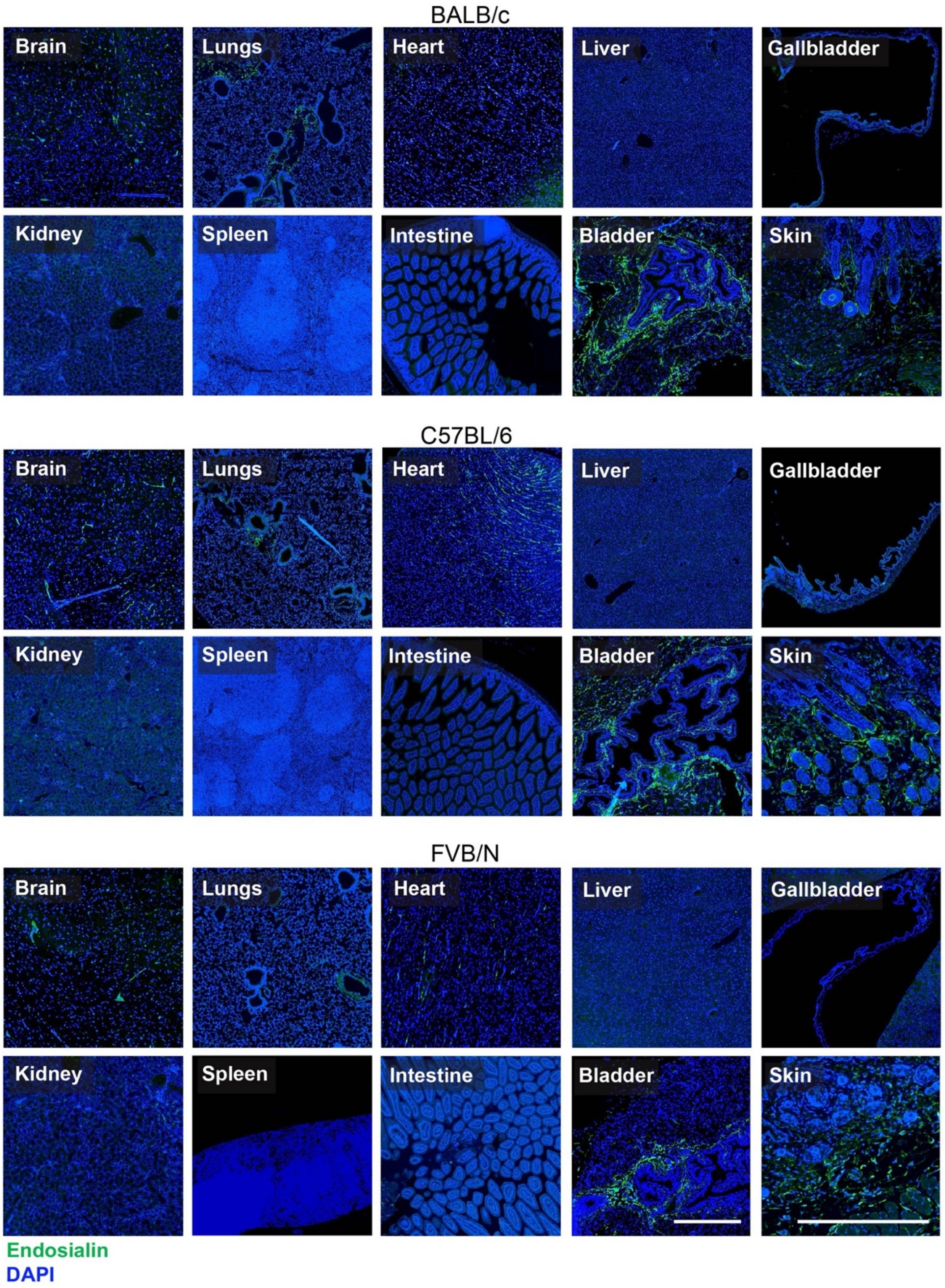

Supplementary Figure 1. Endosialin expression in healthy adult mouse tissues.
Tissue sections from 8-week-old BALB/c (upper panel), C57BL/6 (center panel) and FVB/N (lower panel) mice were stained with the P13 polyclonal anti-endosialin Ab (green) and counterstained with DAPI (blue). Representative images of tissue section from an individual mouse for each strain. Scale bars 500 μm. Skin shown at 2x magnification.

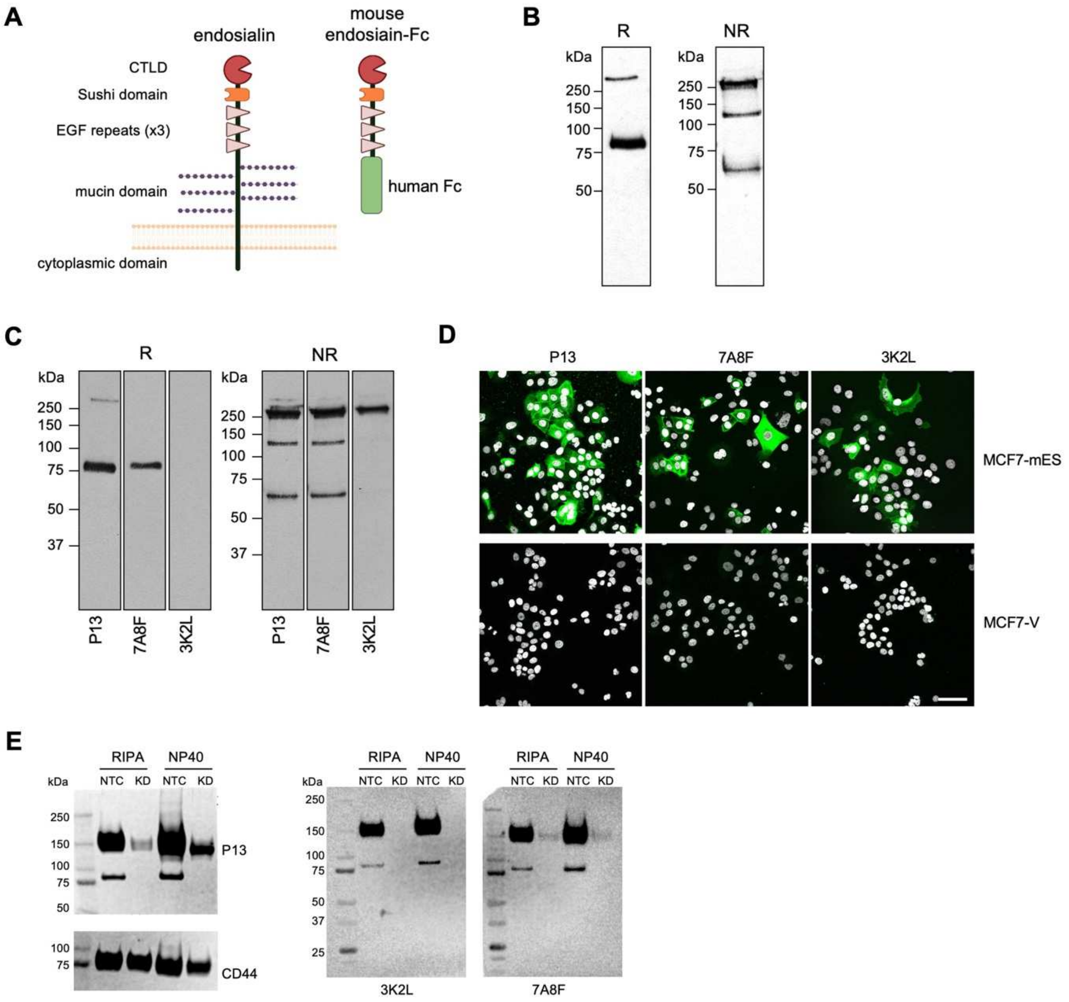

Supplementary Figure 2. Characterization of the rat anti-endosialin 3K2L and 7A8F antibodies.

(A) Diagram showing the domain structure of endosialin and the soluble mouse endosialin-Fc fusion protein. (B) Mouse endosialin-Fc protein was resolved by 10% SDS-PAGE under reducing (R) and non-reducing (NR) conditions and probed with an HRP-conjugated goat anti-human Fc Ig. (C) 0.5 μg of the mouse endosialin-Fc protein was resolved by 10% SDS-PAGE under reducing (R) or non-reducing (NR) conditions and subject to Western blotting with either the polyclonal anti-endosialin P13 antibody followed by HRP-conjugated anti-rabbit Ig or with mAbs 7A8F or 3K2L followed by HRP-conjugated anti-rat Ig. (D) MCF7 human epithelial cells transfected with mouse endosialin (MCF7-mES) or vector alone (MCF7-V) and stained with either the P13 rabbit polyclonal anti-endosialin antibody followed by Alexa488-conjugated anti-rabbit Ig or mAbs 7A8F and 3K2L followed by Alexa488 anti-mouse Ig (green). Nuclei were counterstained with TO-PRO-3 (white). Scale bar, 20 μm. Parts of these images were previously shown in Fig. 1 of MacFadyen et al., 2007[2] and Supplementary Fig. 2 of Simonavicius et al., 2012.[3] (E) 10T1/2 mouse pericyte-like cells were transduced with a non-targeting control shRNA (NTC) or an mouse endosialin shRNA (KD). Cells were lysed in RIPA or NP40 buffers, resolved on non-reducing SDS PAGE gels and probed with P13 polyclonal antibody or mAbs 3K2L and 7A8F following by HRP-conjugated anti-rabbit or anti-rat IgG, respectively. CD44 detected with the IM7 rat mAb was used as a loading control.

Ash SL, et al. J Immunother Cancer 2024; 12:e008608. doi: 10.1136/jitc-2023-008608

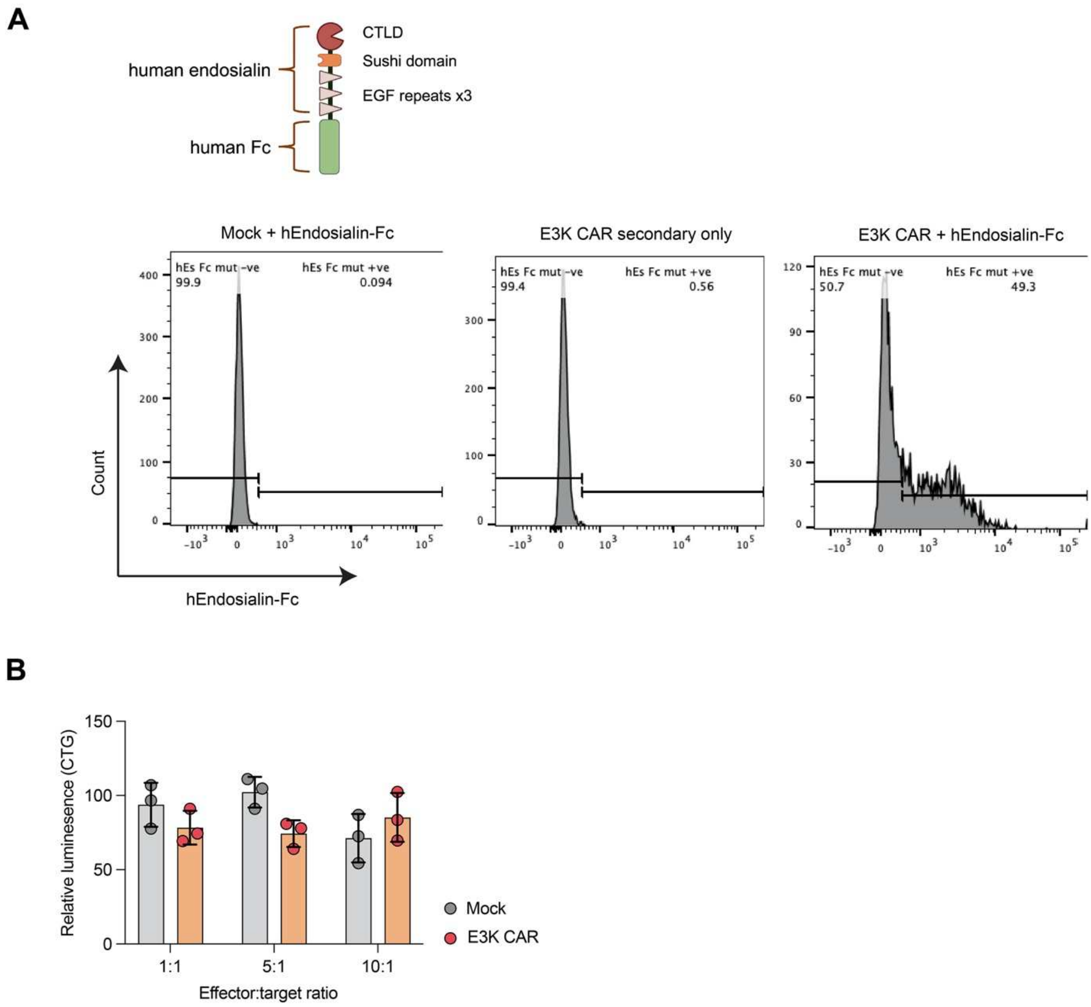

Supplementary Figure 3. E3K CAR-T cells bind endosialin and exhibit no activity against endosialin⁻ 4T1 cells.

(A) BALB/c E3K CAR-T cells were incubated with 1 µg human endosialin-Fc protein (see diagram) for 30 minutes on ice following a CD16.2 block (BioLegend, 149502, clone 9E9, 1:200) and were then subsequently incubated with anti-human IgG_1-AlexaFluor 488. Zombie Violet (1:1000, BioLegend, 423113) was used for live/dead discrimination. Mock T-cells and E3K CAR-T cells incubated without endosialin-Fc protein were used as controls. (B) Cytotoxicity assay of C57/BL6 E3K CAR-T cells against 4T1 cells was performed as described in figure 2D. Data represents triplicate technical repeats (mean values ± SD) in n=1 experiment.

Ash SL, et al. J Immunother Cancer 2024; 12:e008608. doi: 10.1136/jitc-2023-008608

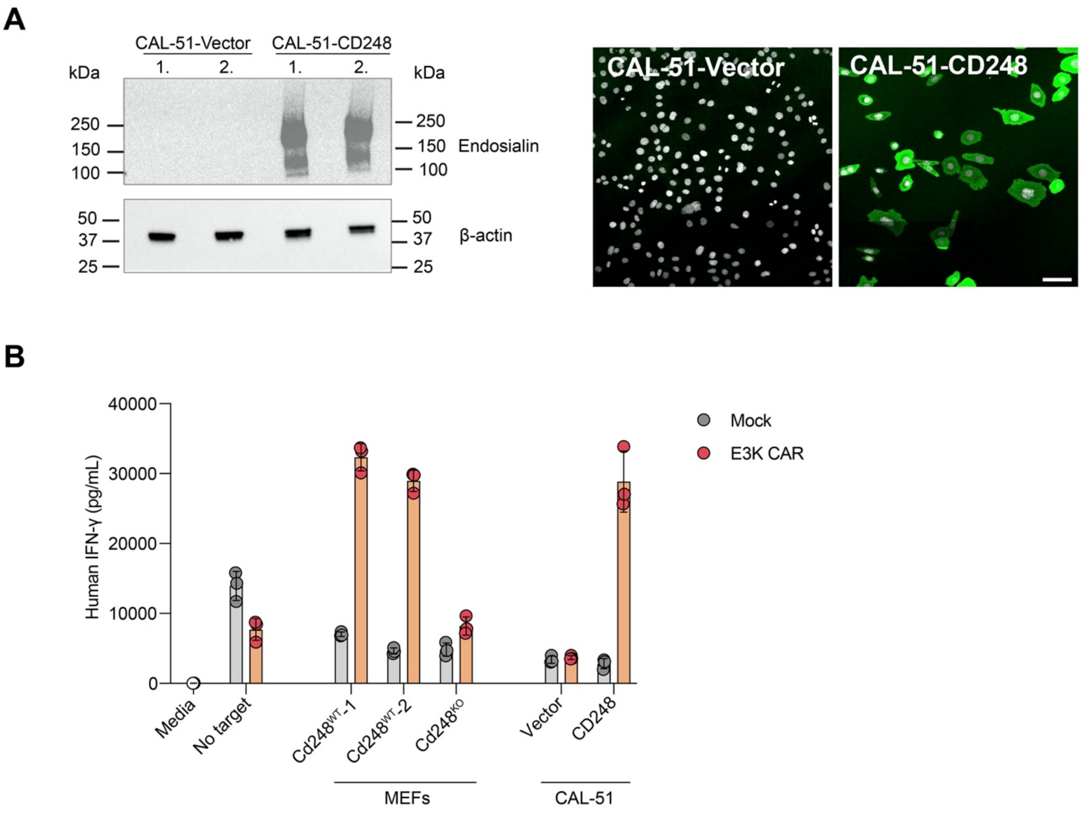

Supplementary Figure 4. Human E3K CAR-T cells recognize and are activated by mouse and human endosialin$^+$, but not endosialin$^-$, cells.

(A) Human CAL-51 breast cancer cells transfected with pCDNA3 vector alone or human *CD248*. Left panel, two independent cell lysates per cell line were run on a non-reducing SDS PAGE gel and blots probed with the anti-human endosialin antibody B1/35. Right panel, cells were stained for endosialin (green), nuclei were counterstained with TO-PRO-3 (white). Scale bar, 20 µm. (B) IFN-γ production by human Mock and E3K CAR-T cells in response to mouse and human endosialin. Transduced T cells were co-cultured with *Cd248*^{WT}, endosialin-expressing mouse embryonic fibroblasts (MEFs): *Cd248*^{WT}; 1 and 2 denote two independent MEF cultures) or MEFs from endosialin knockout mice (*Cd248*^{KO}); or with vector alone transfected CAL-51 cells and CAL-51 cells engineered to express human *CD248*. Target cells were plated at $1x10^4$ cells/well, 48h prior to the addition of $1x10^5$ human T cells/well and after 18 hours, human IFN-γ was detected in culture supernatants by ELISA (see Supplementary Methods for assay details). Data shown (mean values ± SD).

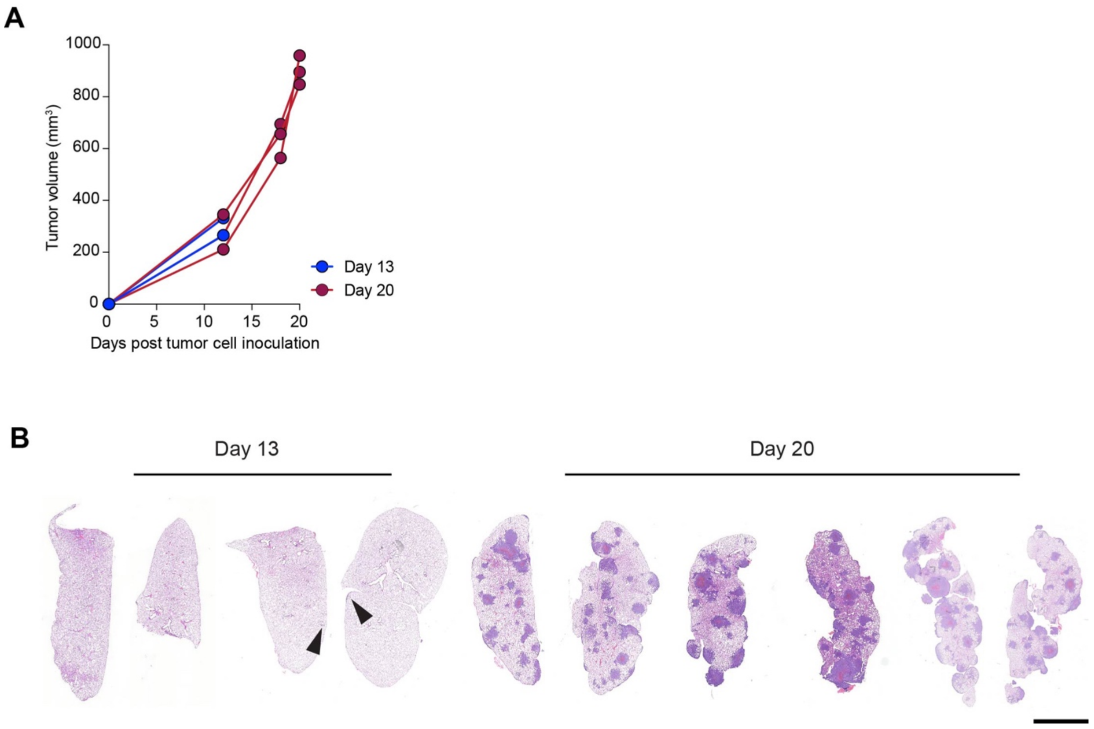

Supplementary Figure 5. 4T1 tumor growth in NSG mice.
Associated with figure 3. 2.5 x 10^5 4T1-Luc cells were inoculated subcutaneously into 5 NSG mice. Two mice were sacrificed on day 13, three mice were sacrificed on day 20. (A) Individual tumor growth curves. (B) H&E-stained lung sections from all mice. Arrowheads indicate small metastatic lesions. Scale bar, 2.5 mm.

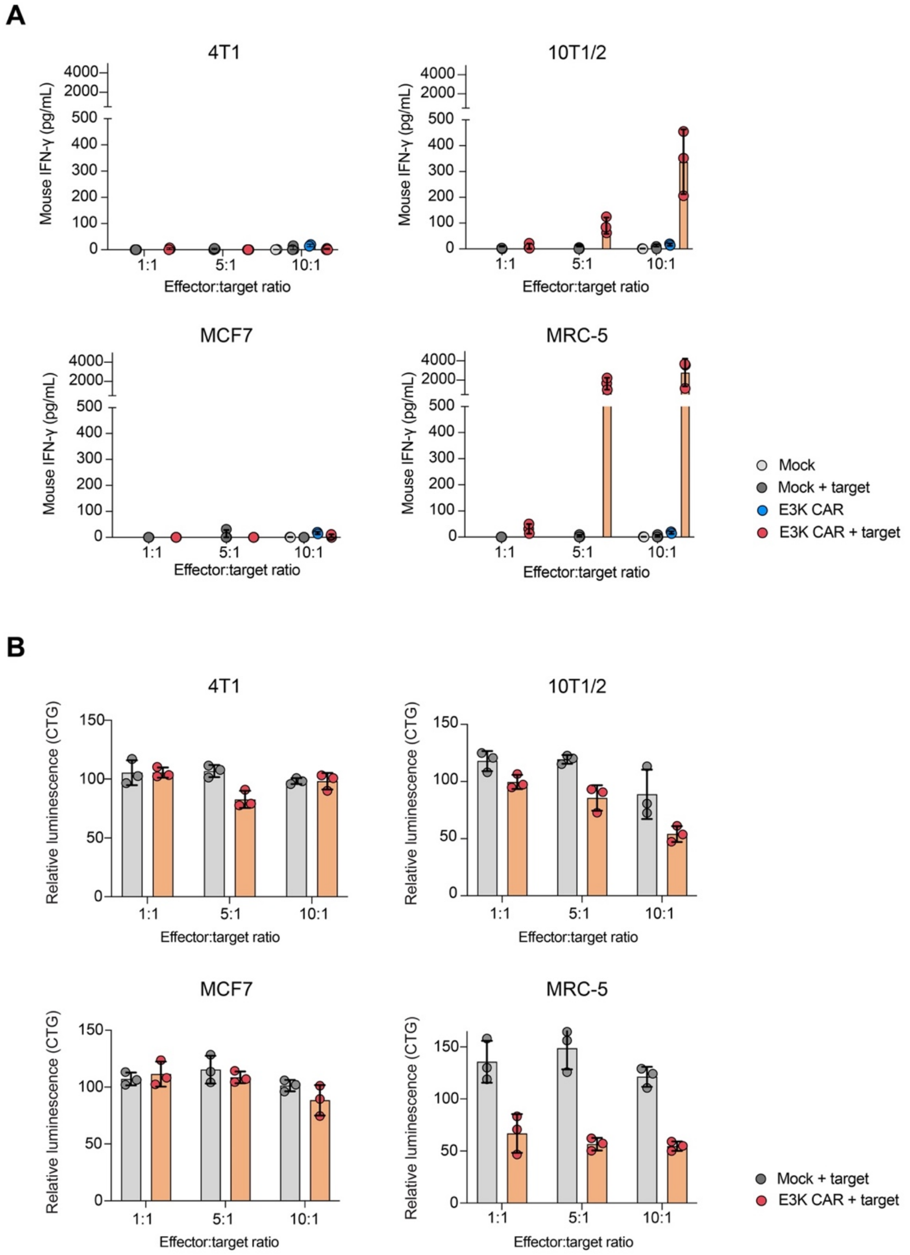

Supplementary Figure 6. *In vitro* characterization of BALB/c E3K CAR-T cells.
Associated with figure 4. *In vitro* activity of BALB/c Mock and E3K CAR-T cells against endosialin+ mouse 10T1/2 and human MRC-5 fibroblasts and endosialin- mouse 4T1 and human MCF7 tumor cells was assessed as described in figure 2D. (A) IFN-γ release into culture supernatants. Data shown (mean values ± SD of technical repeats) is representative of n=2 (human lines) or n=3 (mouse lines) independent assays. (B) CellTiter-Glo (CTG) of target cells was assessed after 96 hours. The average read-out from target cells cultured alone was defined as 100% viability. Data shown (mean values ± SD for technical repeats) is representative of n=3 (human lines) or n=4 (mouse lines) independent assays.

Ash SL, *et al. J Immunother Cancer* 2024; 12:e008608. doi: 10.1136/jitc-2023-008608

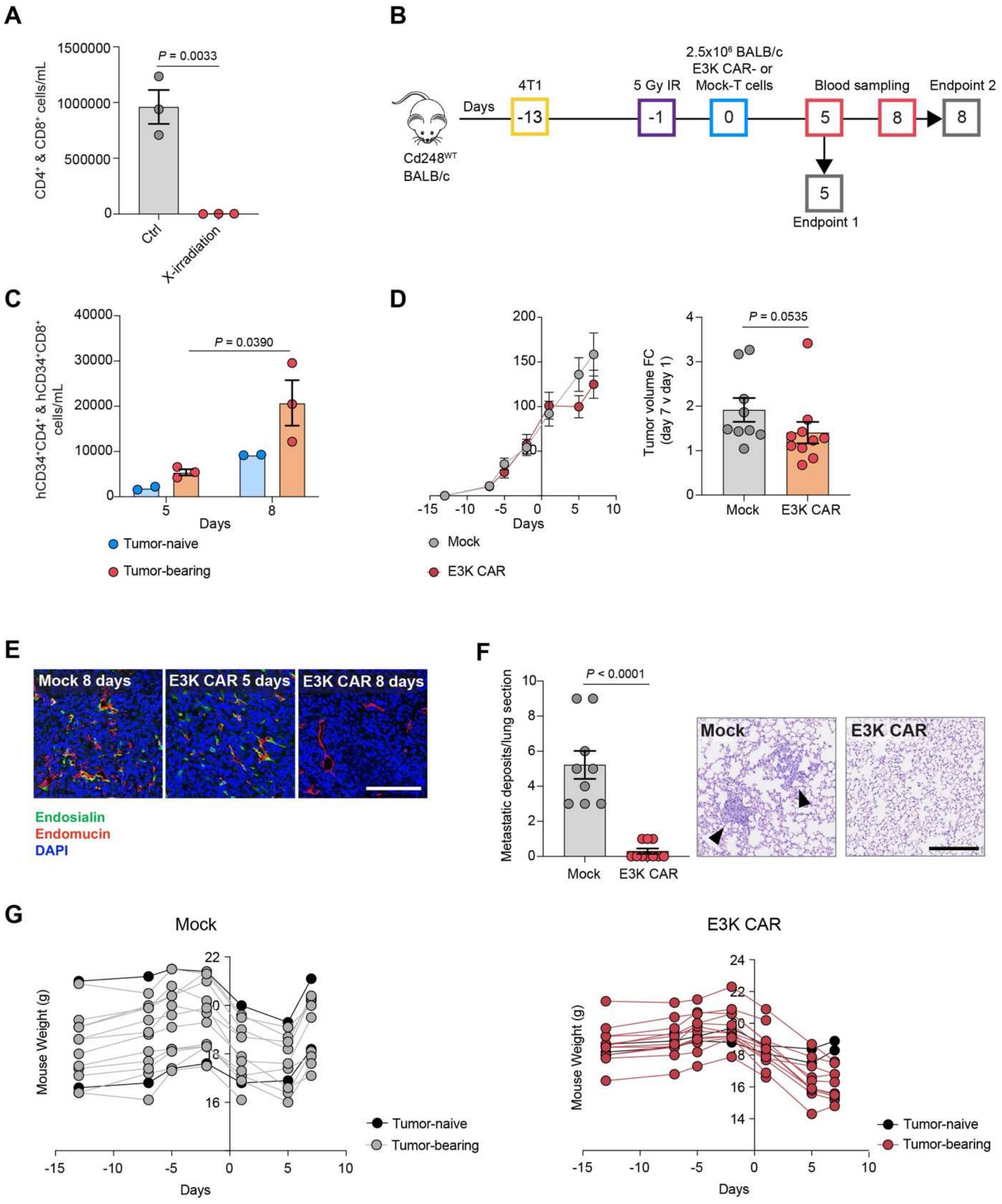

Supplementary Figure 7. Characterization of E3K CAR T-cell activity in a syngeneic BALB/c model.

Associated with figure 4. (A) BALB/c mice were left unirradiated (Ctrl) or subjected to 5 Gy X-irradiation (n=3 mice per group), 18 hours later venous blood was collected via the tail vein. Shown are the combined number of circulating CD4$^+$ and CD8$^+$ T cells per mL (mean values ± SEM; unpaired *t*-test). (B) Experimental timeline of ACT. BALB/c mice were inoculated subcutaneously with 2.5 x 10^5 4T1-Luc cells. Tumors were allowed to grow for 12 days to 40-200 mm^3 before mice were subjected to 5 Gy X-irradiation 18 hours prior to intravenous injection of BALB/c Mock or E3K CAR-T cells (n=11 and n=12 Mock and E3K CAR tumor-

Ash SL, *et al. J Immunother Cancer* 2024; 12:e008608. doi: 10.1136/jitc-2023-008608

bearing mice per group, respectively; n=2 tumor-naive mice per group). Of the tumor-bearing mice, two mice per group were sacrificed on day 5 post-ACT, the remaining mice were sacrificed 8 days post-ACT. (C) Circulating combined $hCD34^{+}CD4^{+}$ and $hCD34^{+}CD8^{+}$ CAR-T cells monitored in venous blood from a subset of mice (mean values ± SEM, unpaired *t*-test). (D) Left panel, tumor growth curves. Right panel, fold change in tumor volume from day 1 to day 7 (mean values ± SEM, Mann Whitney *U* test), excluding those sacrificed early (day 5). (E) Representative tumor sections from mice sacrificed on day 5 or 8 stained for endosialin (green), endomucin (red) and counterstained with DAPI (blue). Scale bar, 150 µm. (F) Quantification of lung metastasis from H&E-stained lung sections at day 8 (mean values ± SEM, unpaired *t*-test) and representative H&E-stained sections, arrowheads indicate metastatic lesions. Scale bar, 250 µm. (G) Body weights of individual mice.

Ash SL, *et al. J Immunother Cancer* 2024; 12:e008608. doi: 10.1136/jitc-2023-008608

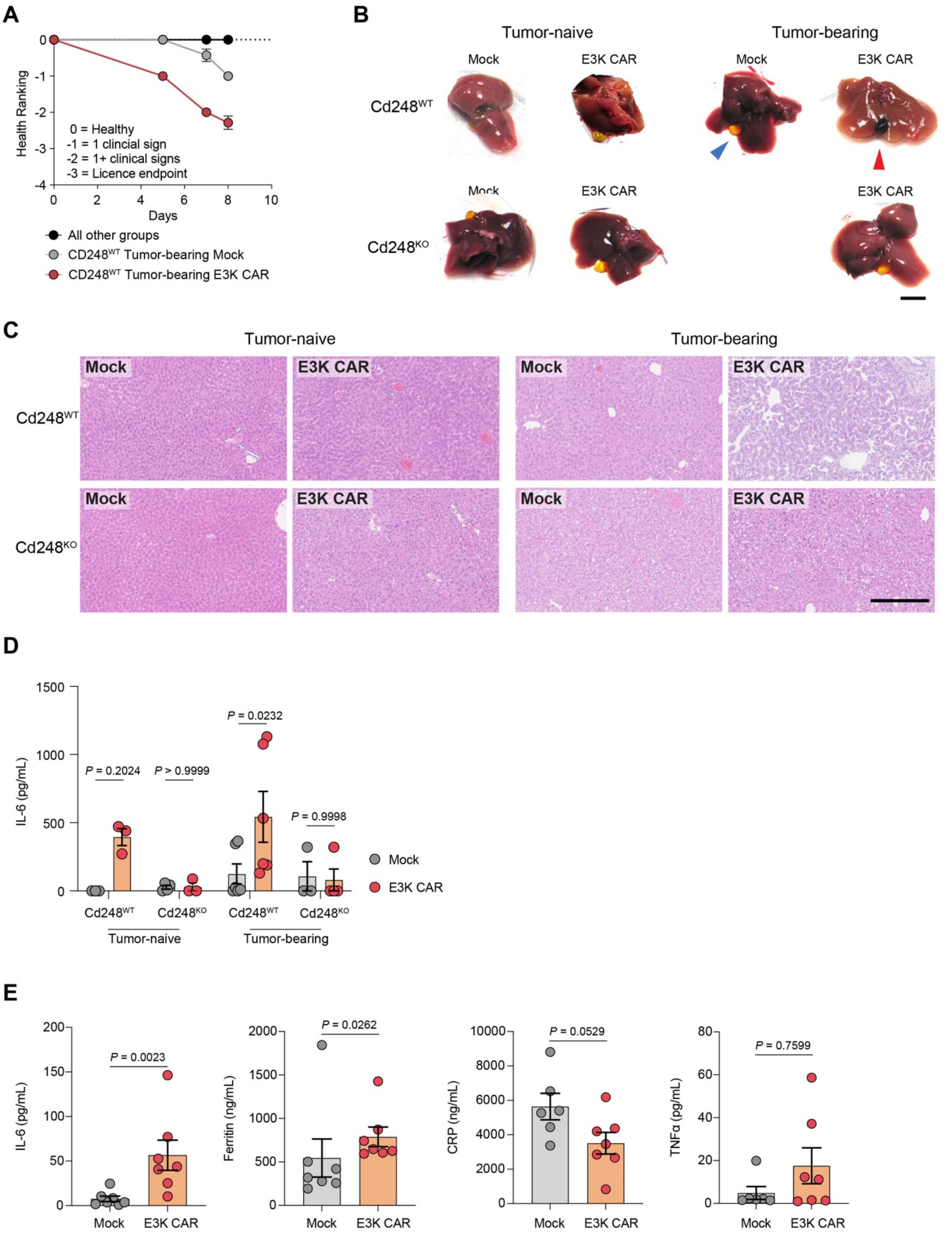

Supplementary Figure 8. E3K CAR-T cell associated toxicity in BALB/c mice is limited to *Cd248*^{WT}, 4T1 tumor-bearing mice.

(A-D) Additional data for figure 4. (A) Health score progression of mice in all groups from figure 4B (see methods; mean values ± SEM). (B) Representative images of liver and attached gallbladders taken at necropsy from all groups apart from Mock-T cell-treated *Cd248*^{KO} tumor-

bearing cohort (photo not taken). Blue arrowhead shows normal gallbladder and red arrowhead shows darkened gallbladder in the *Cd248*^{WT}, 4T1 tumor-bearing mice. Scale bar, 1 cm. (C) Representative H&E-stained liver sections from all groups at endpoint. Scale bar, 250 µm. (D) Mouse IL-6 was measured in the serum of a subset of mice at endpoint by ELISA assay (mean values ± SEM, two-way ANOVA). (E) In an independent experiment a cohort of control *Cd248*^{WT} BALB/c mice were treated with $2.5x10^6$ BALB/c Mock or E3K CAR-T cells. ACT was performed as in figure 4B and E3K CAR-T cell-treated mice were sacrificed 10-14 days post-ACT when symptoms of E3K CAR-related toxicity became apparent, with Mock-T cell-treated mice taken as matched timepoint controls (n=7 for each group). Serum markers were measured at endpoint by ELISA. CRP, C-reactive protein; TNFα, tumor necrosis factor α. Data shows mean values ± SEM, Mann Whitney *U* test apart from CRP (unpaired *t*-test). One Mock sample lost from CRP and TNFα analysis.

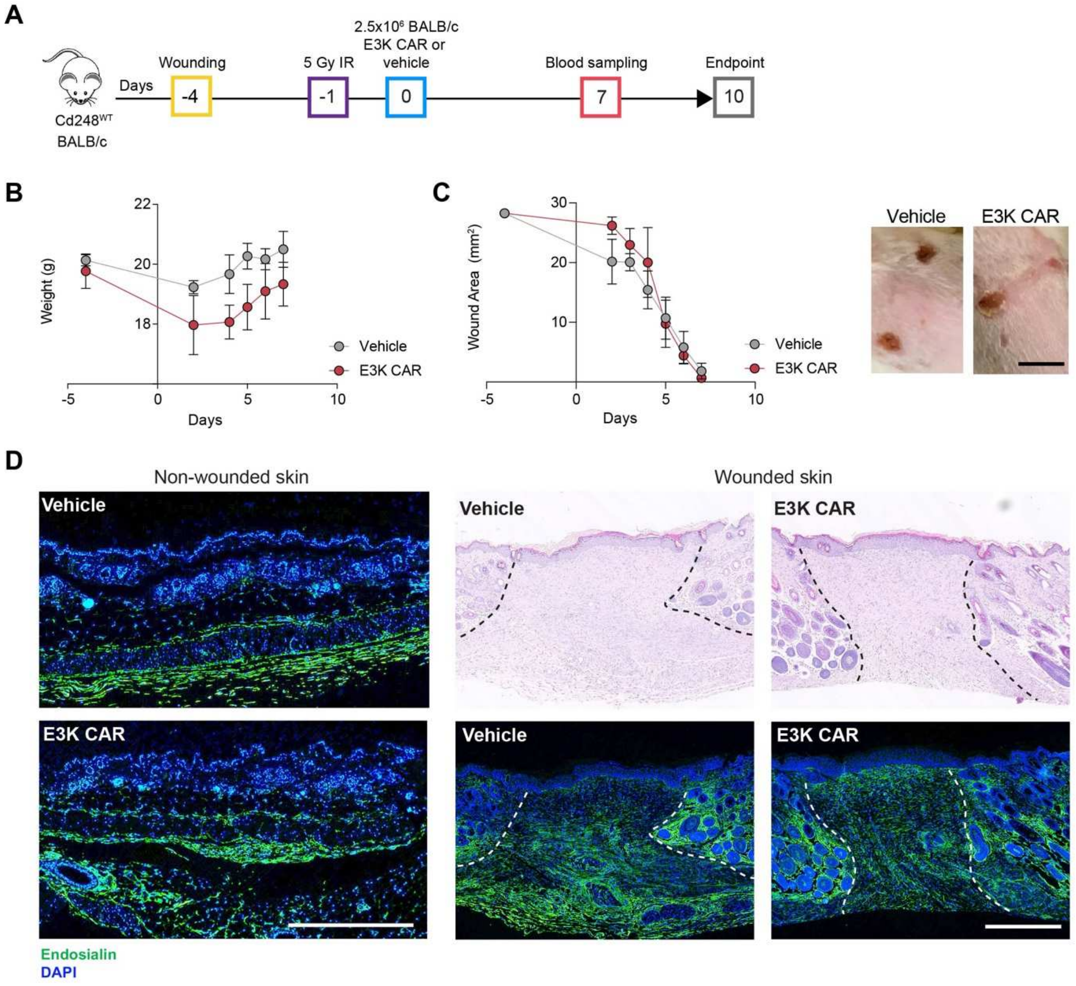

Supplementary Figure 9. E3K CAR-T cells exhibit no activity and are not associated with toxicity in wound healing assays.

(A) ACT experimental timeline. Non-tumor bearing BALB/c mice (n=3 per group) were anesthetized and given 6 mm punch wounds bilaterally using a biopsy punch through the dorsolateral skin. Wounds were protected with sterilized silicon splints as described in (Tan and Wahli 2013.[4] Four days later mice were subject to 5 Gy cesium irradiation (day -1) 18 hours before intravenous injection with 2.5 x 10^6 BALB/c E3K CAR-T cells or vehicle alone (day 0). Mice were sacrificed 10 days post-ACT. (B) Mouse body weights (mean values ± SEM). (C) Wound area of vehicle or E3K CAR-T cell treated mice over time determined from aerial photos of mice taken in the same scaling box (mean values ± SEM). Right panel, representative images of healing wounds 4 days post-ACT. Scale bar, 1 cm. (D) FFPE sections of non-wounded (taken from the flank away from the wound site) and wounded skin tissue taken at endpoint and either H&E-stained or stained with endosialin (green) and counterstained with DAPI (blue). Representative images of non-wounded skin (left panels) and wounded skin (right panels). Dotted lines indicate wound margins. Scale bars, 500 μm.

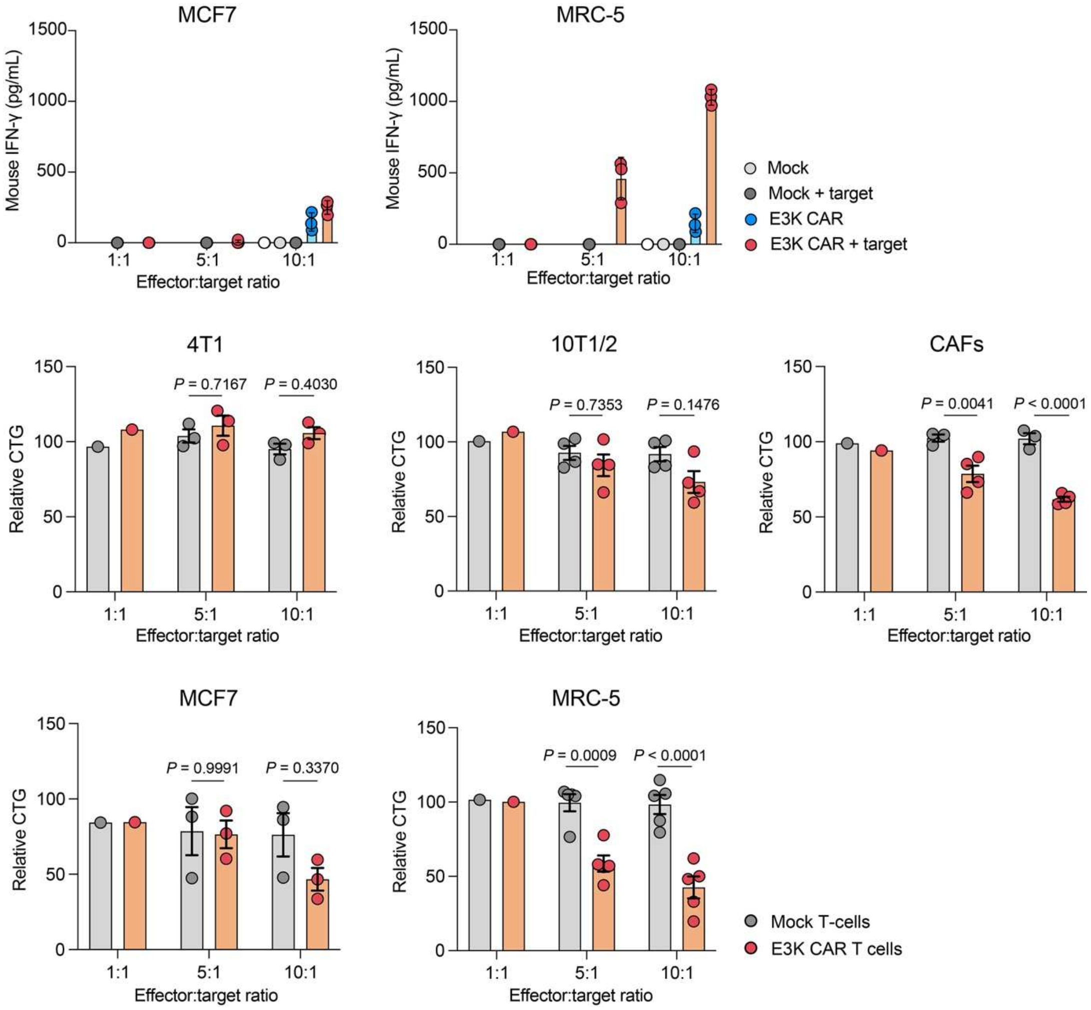

Supplementary Figure 10. *In vitro* characterization of FVB/N E3K CAR-T cells.

Associated with figure 5. *In vitro* activity of FVB/N Mock and E3K CAR-T cells against endosialin$^+$ and endosialin$^-$ target cells was assessed as described in figure 2D, with 1000 mouse or 5000 human target cells seeded per well in 96-well plates. Upper panel, IFN-γ release from co-culture with endosialin$^+$ human MRC-5 fibroblasts and endosialin$^-$ human MCF7 tumor cells. Data shown (mean values ± SD of technical repeats) is representative of n=3 (MCF7) or n=5 (MRC-5) independent assays. Lower two panels, endosialin$^+$ mouse 10T1/2 and human MRC-5 fibroblasts and mouse CAFs, and endosialin$^-$ mouse 4T1 and human MCF7 tumor cells. CellTiter-Glo (CTG) of target cells assessed after 96 hours (mean values ± SEM, two-way ANOVA, n=3-5 independent assays for 5:1 and 10:1 ratios, n=1 independent assay for 1:1 ratio, a minimum of three technical repeats/condition for each assay).

Ash SL, *et al. J Immunother Cancer* 2024; 12:e008608. doi: 10.1136/jitc-2023-008608

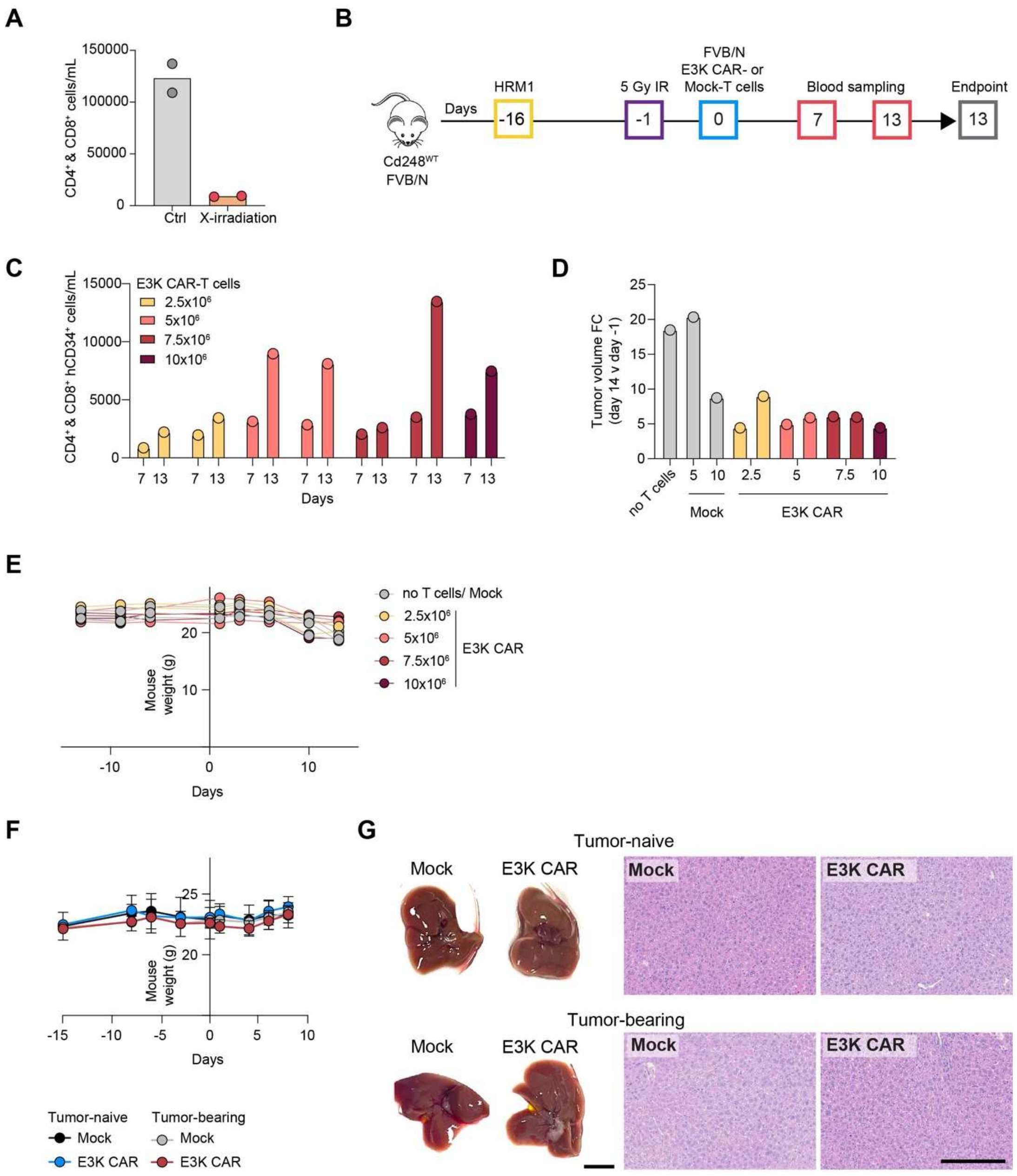

Supplementary Figure 11. Characterization of E3K CAR-T cell activity against HRM1 tumors in FVB/N mice.

(A-F) associated with figure 5. (A) FVB/N mice were left unirradiated (Ctrl) or subjected to 5 Gy X-irradiation (n=2 mice per group). 18 hours later venous blood was collected via the tail vein. Shown are combined number of circulating $CD4^+$ and $CD8^+$ T cells per mL. Data points show individual mice. (B) ACT experimental timeline. *Cd248*^{WT} FVB/N mice were inoculated orthotopically (4th mammary fat pad) with $2x10^5$ HRM1 cells. After 15 days when tumors were 70-250 mm^3, mice were subjected to 5 Gy whole body IR 18 hours prior to injection with 5 or

$10x10^6$ FVB/N Mock (n=1), 2.5-$10x10^6$ E3K CAR-T cells (n=2 apart from $10x10^6$, n=1), or HBSS only (no T cells; n=1). Mice were sacrificed 13 days post-ACT. (C) Circulating combined $hCD34^+CD4^+$ and $hCD34^+CD8^+$ CAR-T cells were monitored in venous blood on day 7 and 13. Paired bars show individual mice for each timepoint. (D) Fold change in tumor volume from day -1 to endpoint. Bars show individual mice. (E) Body weights of individual mice. (F,G) Additional data for figure 5. (F) Body weight of Mock-T and E3K CAR-T cell treated tumor-bearing and non-tumor-bearing mice from figure 5 (mean values ± SEM). (G) Representative images of livers (left panel, scale bar, 1 cm) and H&E-stained liver sections (right panel, scale bar, 250 µm) from tumor-naive and tumor-bearing treated mice at endpoint.

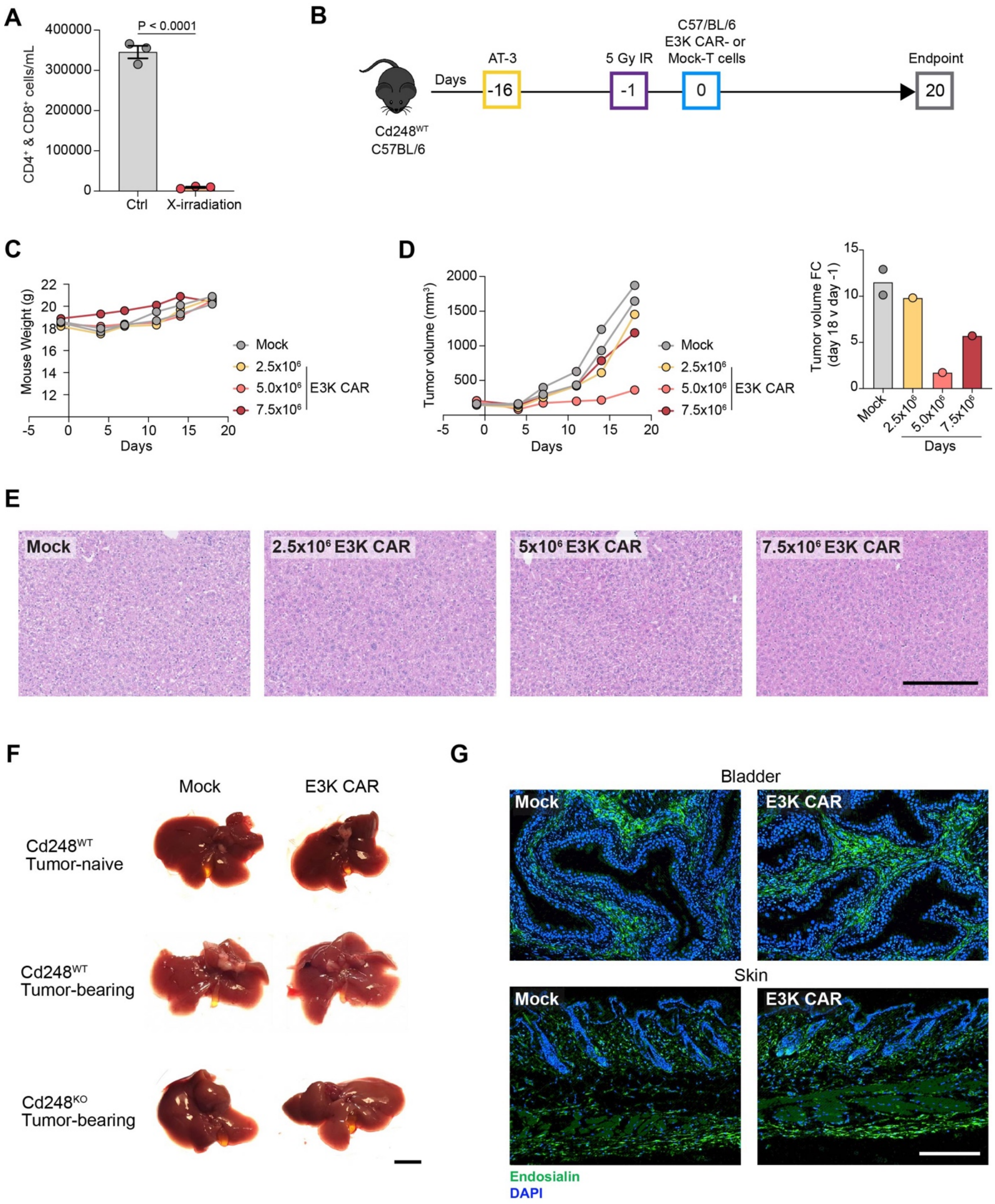

Supplementary Figure 12. Characterization of E3K CAR-T cell activity against AT-3 tumors in C57BL/6 mice.

(A-E) Associated with figure 7. (A) C57BL/6 mice were left unirradiated (Ctrl) or subjected to 5 Gy X-irradiated (n=3 mice per group). 18 hours later venous blood was collected via the tail vein. Shown are the combined numbers of $CD4^+$ and $CD8^+$ T cells per mL (mean values ± SEM, unpaired *t*-test). (B-E) Tumors were allowed to grow for 16 days to 140-270 mm^3 volume. All mice were subjected to 5 Gy whole body X-irradiation 18 hours prior to intravenous injection with 2.5, 5 or 7.5 x 10^6 C57BL/6 E3K CAR-T cells (n=1 per group) or 7.5 x 10^6 C57BL/6 Mock-T cells (n=2). Mice were sacrificed 20 days post-ACT when the first Mock-T cell treated tumor

approached maximum allowable size. (B) Experimental timeline of ACT. *Cd248*^{WT} C57BL/6 mice were inoculated subcutaneously with 2.5 x 10^5 AT-3 cells. (C) Individual mouse body weights. (D) Left panel, tumor growth curves in individual mice. Right panel, fold change in tumor volume from day -1 to day 18. (E) Representative images of H&E-stained liver sections. Scale bar, 250 µm. (F,G) Additional data for the experiment shown in figure 6. Representative images of tissues taken at endpoint from *Cd248*^{WT} tumor-naive and tumor-bearing mice and *Cd248*^{KO} tumor-bearing mice treated with Mock or E3K CAR-T cells. Panel F, livers and attached gallbladders. Scale bar, 1 cm. Panel G, bladder and skin sections from *Cd248*^{WT} tumor-bearing mice treated with Mock or E3K CAR-T cells stained for endosialin (green) and counterstained with DAPI (blue). Scale bar, 250 µm.

Ash SL, *et al. J Immunother Cancer* 2024; 12:e008608. doi: 10.1136/jitc-2023-008608

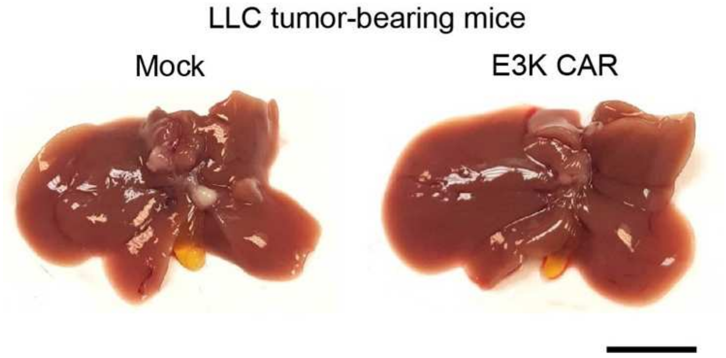

Supplementary Figure 13. E3K CAR-T cells show no toxicity in LLC tumor-bearing mice. Additional data for figure 7. Representative images of livers and attached gallbladders taken at endpoint from *Cd248*^{WT} LLC tumor-bearing mice treated with Mock or E3K CAR-T cells. Scale bar, 1 cm.

References

1. Cheadle EJ, Sheard V, Rothwell DG, et al. Differential Role of Th1 and Th2 Cytokines in Autotoxicity Driven by CD19-Specific Second-Generation Chimeric Antigen Receptor T Cells in a Mouse Model. *The Journal of Immunology* 2014;192(8):3654-65. doi: 10.4049/jimmunol.1302148
2. MacFadyen J, Savage K, Wienke D, et al. Endosialin is expressed on stromal fibroblasts and CNS pericytes in mouse embryos and is downregulated during development. *Gene Expr Patterns* 2007;7(3):363-9. doi: 10.1016/j.modgep.2006.07.006 [published Online First: 20060727]
3. Simonavicius N, Ashenden M, van Weverwijk A, et al. Pericytes promote selective vessel regression to regulate vascular patterning. *Blood* 2012;120(7):1516-27. doi: 10.1182/blood-2011-01-332338 [published Online First: 2012/06/29]
4. Tan NS, Wahli W. Studying Wound Repair in the Mouse. *Curr Protoc Mouse Biol* 2013;3(3):171-85. doi: 10.1002/9780470942390.mo130135 [published Online First: 20131018]
