# Supplementary material for: Targeting the activated microenvironment with endosialin (CD248)-directed CAR-T cells ablates perivascular cells to impair tumor growth and metastasis
Source: J Immunother Cancer. 2024 Feb 27;12(2):e008608. doi: 10.1136/jitc-2023-008608 (PMC10900351; doi:10.1136/jitc-2023-008608)
Supplement: Supplementary data [file jitc-2023-008608supp002.pdf]

Targeting the activated microenvironment with endosialin (CD248)-directed CAR-T cells ablates perivascular cells to impair tumor growth and metastasis

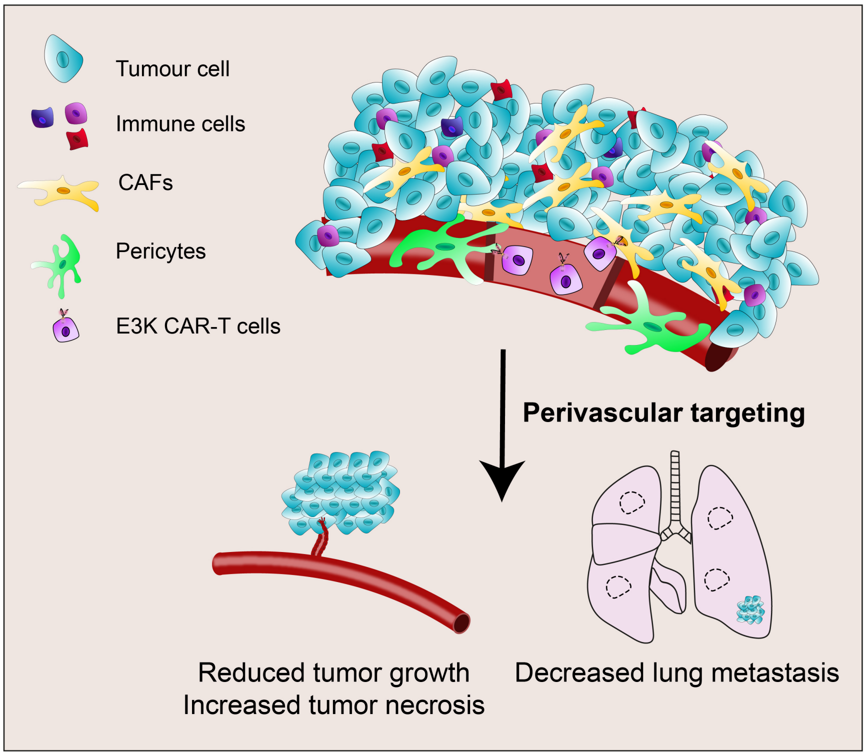

Authors

Sarah L. Ash, Rebecca Orha, Holly Mole, Meg Dinesh-Kumar, Steven P. Lee, Frances K. Turrell, Clare M. Isacke

Correspondence

frances.turrell@manchester.ac.uk, clare.isacke@icr.ac.uk

In brief

Endosialin-directed CAR-T cells target perivascular cells within the tumor stroma impairing disease progression in multiple syngeneic solid tumor models.
